# Sub-picosecond charge-transfer at near-zero driving force in polymer:non-fullerene acceptor blends and bilayers

**DOI:** 10.1038/s41467-020-14549-w

**Published:** 2020-02-11

**Authors:** Yufei Zhong, Martina Causa’, Gareth John Moore, Philipp Krauspe, Bo Xiao, Florian Günther, Jonas Kublitski, Rishi Shivhare, Johannes Benduhn, Eyal BarOr, Subhrangsu Mukherjee, Kaila M. Yallum, Julien Réhault, Stefan C. B. Mannsfeld, Dieter Neher, Lee J. Richter, Dean M. DeLongchamp, Frank Ortmann, Koen Vandewal, Erjun Zhou, Natalie Banerji

**Affiliations:** 1 Department of Chemistry and Biochemistry, University of Bern, Freiestrasse 3, CH-3012 Bern, Switzerland; 2 Chinese Academy of Sciences (CAS) Key Laboratory of Nanosystem and Hierarchical Fabrication, CAS Center for Excellence in Nanoscience, National Center for Nanoscience and Technology, Beijing, 100190 P. R. China; 3 Instituto de Física de São Carlos (IFSC), Universidade de São Paulo (USP), Av. Trabalhador saocarlense, 400, CEP, 13560-970 São Carlos, Brazil; 4 Dresden Integrated Center for Applied Physics and Photonic Materials (IAPP) and Institute for Applied Physics Technische Universität Dresden, Nöthnitzer Str. 61, 01187 Dresden, Germany; 5 Institute of Physics and Astronomy, University of Potsdam, Karl-Liebknecht-Str. 24-25, 14476 Potsdam-Golm, Germany; 6 Material Measurement Laboratory, National Institute of Standards and Technology (NIST), Gaithersburg, MD 20899 USA; 7 Center for Advancing Electronics Dresden, Technische Universität Dresden, Helmholtzstr. 18, 01062 Dresden, Germany; 8 Institute for Materials Research (IMO-IMOMEC), Hasselt University, Wetenschapspark 1, 3590 Diepenbeek, Belgium

**Keywords:** Electron transfer, Solar cells

## Abstract

Organic photovoltaics based on non-fullerene acceptors (NFAs) show record efficiency of 16 to 17% and increased photovoltage owing to the low driving force for interfacial charge-transfer. However, the low driving force potentially slows down charge generation, leading to a tradeoff between voltage and current. Here, we disentangle the intrinsic charge-transfer rates from morphology-dependent exciton diffusion for a series of polymer:NFA systems. Moreover, we establish the influence of the interfacial energetics on the electron and hole transfer rates separately. We demonstrate that charge-transfer timescales remain at a few hundred femtoseconds even at near-zero driving force, which is consistent with the rates predicted by Marcus theory in the normal region, at moderate electronic coupling and at low re-organization energy. Thus, in the design of highly efficient devices, the energy offset at the donor:acceptor interface can be minimized without jeopardizing the charge-transfer rate and without concerns about a current-voltage tradeoff.

## Abstract (web summary)

It has been commonly believed that the driving force at the donor-acceptor heterojunction is vital to efficient charge separation in organic solar cells. Here Zhong et al. show that the driving force can be as small as 0.05 eV without compromising the charge transfer rate and efficiency.

## Introduction

Organic heterojunctions between electron donors (D) and acceptors (A) are of vital importance for diverse applications ranging from photocatalysis, to batteries and solar energy conversion^1–3^. Appropriately selected D:A combinations have enabled high performance in organic electronic devices such as organic photovoltaics (OPVs) or organic light-emitting diodes (OLEDs)^3,4^. Future optimization of the fundamental optoelectronic processes occurring at the heterojunctions of OPV systems crucially relies on measuring and understanding the dynamics of photogenerated interfacial species. Typically, excitons dissociate at the D:A heterojunction by charge-transfer (CT) processes and subsequently separate into free charges, which are extracted as photocurrent. Common electron transfer theories predict that the driving force, namely the free energy difference between the CT state and the photoexcited (S_1_) state, is a key factor determining the rate of the CT^5–9^. In OPV systems, a dependence of the CT rate and yield on the driving force has indeed been observed for fullerene-containing model systems^10,11^. This is consistent with the previously reported empirical lower limit of a 0.3 eV driving force required for efficient CT in typical polymer:fullerene blends^12,13^. Understanding the impact of driving force on the CT dynamics is particularly important in organic solar cells, since the CT state energy also determines the open circuit voltage (*V*_OC_), so that a small driving force is desirable, but might lead to a tradeoff in current generation if the recombination of excitons competes with their slow dissociation^13–17^.

In this respect, the recent success of non-fullerene acceptors (NFAs), that have taken the OPV community by storm with record device efficiencies over 16%^3,9,18–23^, has been attributed to efficient current generation in polymer:NFA blends even at very low or absent driving force, reducing *V*_OC_ losses^3,19,24^. Moreover, NFAs show high absorption in the visible and near-infrared (NIR) range, which can be complemented with the donor absorption to cover a broad spectrum for light harvesting. For a further optimization of the power conversion efficiency, it is now essential to understand how the charge generation dynamics are impacted by acceptor light harvesting and the low driving force for CT, which both distinguish the NFA systems from fullerene blends. Several reports on polymer:NFA systems claim a reduced CT rate on the picosecond time scale, attributed to the low driving force^15,16,25,26^. There are however two major shortcomings in these studies. First, the investigations have not clearly established whether the observed CT rates reflect only interfacial processes or whether they are limited by exciton diffusion in the complex morphology of the investigated blends^15,16,25,27–29^. Second, awareness must be raised that charges in NFA systems are generated by distinct ET or HT channels, with different driving forces and possibly different rates.

In this work, we have carried out a carefully designed transient absorption (TA) study on polymer:NFA systems. We disentangle intrinsic CT rates from morphological aspects (determined using X-ray diffraction techniques) by comparing the HT rate of the optimized blend with the corresponding planar heterojunction (bilayer) system and a dilute blend containing a low NFA concentration. We show that in the absence of exciton diffusion, HT occurs on the sub-picosecond time scale in spite of a negligible ≈0.05 eV driving force, which we establish using sensitive external quantum efficiency (sEQE) and electroluminescence (EL) measurements. We find that in blends of the same NFA component mixed with different polymers, the intrinsic HT time (inverse rate) decreases from 400 to 80 fs when the driving force increases from 0.05 to 0.4 eV. The behavior at low driving force is consistent with the trend expected in the Marcus normal region for CT with moderate electronic coupling, whereby the high rates can be explained by a small reorganization energy. Using DFT calculations, we find indeed moderate electronic coupling (21 meV) and low reorganization energy (161 meV) for HT in our highest-efficiency system. Moreover, we demonstrate that HT is generally slower (<1 ps) than ET (<0.1 ps) at comparable driving force, likely related to a higher transfer integral (electronic coupling) for ET. The ultrafast ET rates can no longer be described within the non-adiabatic Marcus limit, as already established for polymer:fullerene blends^30–33^. Overall, the sub-picosecond times for both CT pathways over a large range of driving forces demonstrate that the energy offset at the heterojunction can be minimized without jeopardizing the CT rate and efficiency. This positive message eliminates the concern about current–voltage tradeoffs in the future design of highly efficient non-fullerene solar cells with low driving force.

## Results

### Material systems

**Fig. 1:**
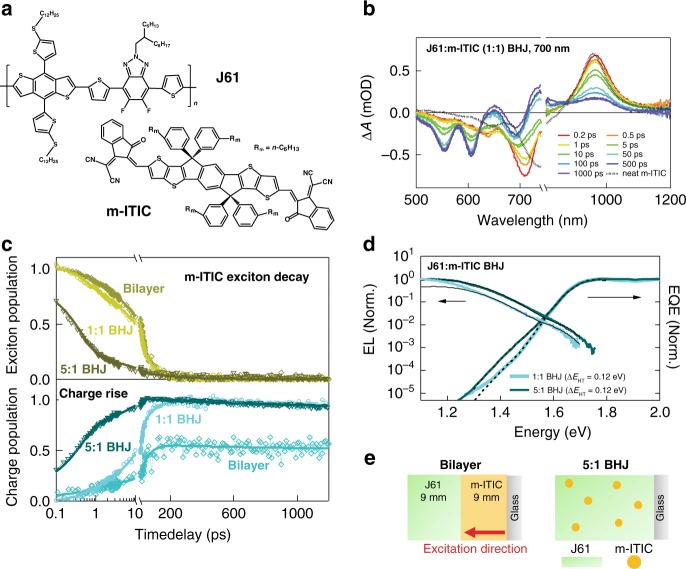
HT in J61:m-ITIC heterojunctions with different sample configurations. **a** Chemical structure of m-ITIC and J61. **b** TA spectra at selected time delays (see legend) recorded for the J61:m-ITIC (1:1) BHJ following excitation at 700 nm. **c** m-ITIC exciton decay (top) and charge rise (bottom) dynamics for J61:m-ITIC samples under different morphological scenarios, upon selective m-ITIC excitation at 700–730 nm, obtained from the analysis of the TA data. The *y*-axis is expressed as a fraction of the total absorbed photon density. Symbols are the experimental data and solid lines are exponential fits obtained globally for the exciton decay and charge rise. **d** sEQE and EL spectra for the J61:m-ITIC BHJ blends with 1:1 and 5:1 mass ratio. The solid and dashed black lines are fits to the EL and sEQE spectra with bi-Gaussian functions, respectively, yielding the S_1_ and CT energies as global parameters. **e** Schematic illustration of the morphology in the J61:m-ITIC bilayer and dilute (5:1 BHJ) samples.

We select the J61:m-ITIC system (Fig. 1a) for our investigation (see chemical structures of all materials in Supplementary Fig. 1, and full name of these chemicals in the section “Methods”). This system is chosen since (i) the optimized 1:1 (mass ratio) blend shows high OPV efficiency of around 12%^34^, (ii) m-ITIC is representative of the ITIC-based core structure and general acceptor–donor–acceptor backbone motif of highly efficient (over 16%) NFAs^34–36^, (iii) the distinct absorption of the J61 donor and the m-ITIC acceptor allows for selective excitation of either (Supplementary Fig. 2), and (iv) the system has a low driving force for HT leading to high *V*_OC_ of 0.9 V and a typical voltage loss (*E*_CT_−*V*_OC_) of 0.65 eV^34,37^. By combining different donor polymers with m-ITIC, we are able to generalize our conclusions to a broad range of donor:NFA systems. Even if other NFA-based devices with lower voltage loss have been reported (current record PCE over 16% with *V*_OC_ = 0.86 eV and voltage loss = 0.53 eV)^36^, which might show different recombination dynamics, the range of donor:acceptor blends we use here reflects most state-of-the-art NFA systems when addressing the question of how the low driving force affects the HT mechanism.

### Hole transfer dynamics in the J61:m-ITIC blend

In the optimized 1:1 blend under 700 nm excitation of the acceptor, only the signatures of photo-excited m-ITIC (slightly red shifted compared to the neat film) are present in the TA spectra at early time delays and gradually convert to the signatures of charges, as the m-ITIC excitons dissociate by HT (Fig. 1b, Supplementary Figs. 3 and 4). To determine the HT rate, we have decomposed the TA spectra at each time delay into a linear combination of the m-ITIC exciton spectrum and the charge spectrum, using a linear least-square fitting procedure (only two components are needed, see Supplementary Note 1)^38^. The temporal evolution of the two components (Fig. 1c) shows a correlated decay of the excitons and rise of the charges with global time constants of 0.8, 12, and 82 ps (Table 1 and Supplementary Table 1), indicating that the m-ITIC excitons decay primarily by efficient dissociation. In principle, the average HT time of 35 ps agrees with relatively slow HT reported in literature for polymer:NFA systems with low driving force^15,16,29^. However, the weak 0.8 ps (14%) component points to a sub-picosecond intrinsic HT time scale if excitons are generated near a D:A interface or in an intermixed D:A region^39^, while the multiphasic slower charge generation could be limited by exciton diffusion.

**Table 1:** Charge-transfer rates for different driving forces.

Sample	*λ*_ex_ (nm)	*E*_S1_ (eV)	*E*_CT_ (eV)	−Δ*E* (eV)	Rise times of charges (ps)
J61:m-ITIC (1:1)	700	1.67	1.55	0.12	**0.78 (−14%)** 12 (−50%) 82 (−36%)
J61:m-ITIC bilayer	700	1.65	n.a	n.a.	**0.92 (−17%)** 46 (−83%)
PCDTBT:m-ITIC (5:1)	730	1.68	1.63	0.05	**0.40 (−57%**) 13 (−43%)
J61:m-ITIC (5:1)	730	1.68	1.56	0.12	**0.43 (−70%)** 7.8 (−30%)
PBTTT:m-ITIC (5:1)	730	1.70	1.34	0.36	**0.16 (−78%)** 4.0 ps (−22%)
P3HT:m-ITIC (5:1)	730	1.69	1.27	0.42	**0.08 (−92%)** 2.6 ps (−8%)
PCDTBT:m-ITIC (1:1)	480	1.88	1.62	0.26	**<0.06 (−40%)** 0.3 (−35%) 17 (−25%)
J61:m-ITIC (1:1)	480	2.00	1.55	0.45	**<0.06 (−34%)** 0.5 ps (−5%) 8.4 ps (−39%) 57 (−22%)
P3HT:m-ITIC (1:1)	480	2.00	1.20	0.80	**<0.06 (−13%)** 0.5 ps (−18%) 8.7 ps (−69%)

Excited-state energy *E*_S1_ of the acceptor (700 or 730 nm excitation) or donor (480 nm excitation), CT state energy (*E*_CT_) and driving force for exciton splitting (-Δ*E*). The charge rise time constants (in ps) and their weight (%), obtained from the analysis of the TA data, are also shown. The first time constant (bold) corresponds to the intrinsic charge-transfer time.

To verify this hypothesis, we move away from the morphological complexity of the optimized BHJ, to different sample configurations with more structural control, namely a bilayer fabricated by a lamination process^40–46^, and a 5:1 BHJ where the m-ITIC acceptor is present in dilute concentration (TA spectra in Supplementary Fig. 4). In the former, the lamination method has been shown by X-ray reflection and X-ray photoelectron spectroscopy (XPS) to yield a flat and well-defined interface with minimal molecular interdiffusion (lower than 0.01% mass)^46,47^, maintaining the absorption properties of each neat layer (Supplementary Fig. 2) and allowing a precise determination of the interfacial exciton density. In the latter, we aim to disperse the acceptor in the polymer matrix in order to minimize the exciton diffusion before it reaches the heterojunction. Grazing-incidence wide-angle X-ray scattering (GIWAXS) and resonant soft X-ray scattering (R-SoXS) agree with the existence of m-ITIC-rich domains (leading to exciton diffusion) alongside ordered pure J61 and possibly polymer:NFA mixed regions in the 1:1 blend, while the acceptor is largely intermixed within the polymer matrix (which maintains similar aggregation as pure J61) in the 5:1 blend (see Supplementary Note 2 and Supplementary Fig. 5). We use a simultaneous analysis of the reduced EL and sEQE spectra with bi-Gaussian functions (Supplementary Note 3) to find both the S_1_ and CT state energies of the samples (Fig. 1d, Table 1 and Supplementary Table 2). We thus determine that the driving force for HT is relatively low (0.12 eV) and similar in the 1:1 and 5:1 BHJ blends (see bilayer results in Supplementary Fig. 6).

From the TA dynamics (Fig. 1c, Table 1), we find that in the bilayer, the average HT time is slightly slower (38 ps) than in the 1:1 blend due to enhanced exciton diffusion through the 9 nm m-ITIC layer (Fig. 1e), and that not all excitons reach the D:A interface for dissociation (55% charge yield compared to the optimized BHJ). On the other hand, in the 5:1 dilute blend, the charge generation is much faster (2.6 ps on average), evidencing the successful dispersion of m-ITIC in J61 and reduced exciton diffusion (Fig. 1e). Most importantly, we observe the sub-picosecond rise of charges in all three sample, as shown by the fastest time constant of 0.4 (70%), 0.8 (14%), and 0.9 ps (17%) for the 5:1 blend, 1:1 blend, and bilayer, respectively (Table 1). The dilute 5:1 BHJ, in particular, shows that 70% of the charges are formed by HT with an intrinsic time constant (not limited by exciton diffusion) of only 0.4 ps in spite of a low driving force of 0.12 eV. The remaining 30% of the charge rise (in 7.8 ps) could be due to residual aggregation and exciton diffusion in the 5:1 blend, or due to less favorable D:A geometries in the blend. Indeed, DFT calculations show that the electronic coupling and hence the transfer rate dramatically drop when the molecules are slightly further apart than in the equilibrium conformation (see discussion below). The slightly slower onset of HT in the other samples (1:1 blend and bilayer, 0.8 and 0.9 ps) can be explained by a less precise determination of the fastest time constant due to its low weight (14% and 17%), by a different molecular conformation between the donor and acceptor (we expect better coupling when m-ITIC is surrounded by J61 in the dispersed system)^48^, or by the influence of different molecular aggregation on the CT rate^10,49^. Nevertheless, the intrinsic time scale for HT remains surprisingly fast (<1 ps) no matter what phase morphology is present, in sharp contrast to previous observations (HT in ≈about 10 ps), where the influence of exciton diffusion was not accounted for^15,16,26,29^.

### Electron transfer dynamics in the J61:m-ITIC blend

**Fig. 2:**
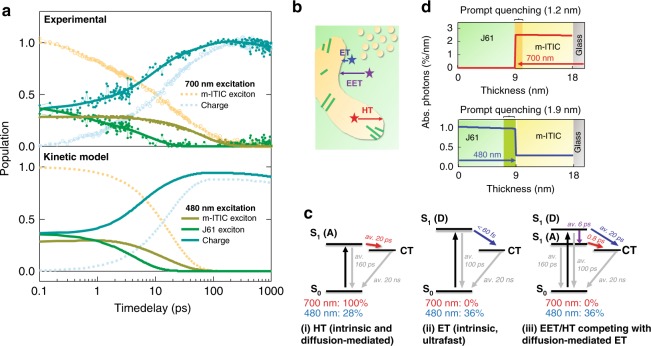
Comparison of the ET and HT processes in J61:m-ITIC. **a** m-ITIC and J61 exciton decay and charge rise dynamics in the J61:m-ITIC (1:1 BHJ) sample under 700 and 480 nm excitation obtained from the analysis of the experimental TA data (top), and corresponding dynamics simulated by kinetic modeling (bottom). **b** Schematic representation of the processes used in the kinetic model within the phase morphology of the blend, which comprises m-ITIC-rich domains (orange), neat ordered polymer domains (green) and intermixed donor–acceptor regions. **c** Jablonski diagram and time constants for the processes described by the kinetic model: i. 100% and 28% of photons are directly absorbed by m-ITIC at 700 and 480 nm, respectively, which then undergoes intrinsic and diffusion-mediated HT; ii. At 480 nm, J61 excitons generated within 1.9 nm of a m-ITIC interface undergo ultrafast ET; iii. J61 excitons generated further from an interface undergo diffusion-mediated ET in competition with EET followed by interfacial intrinsic HT, which is predominant due to a shallower distance dependence of EET. Note that all multiphasic processes are approximated with average time constants, leading to some differences with the experimental data. **d** Excitation profiles (percentage of total incident photons absorbed per nanometer, calculated by TMM) for the bilayer sample at both excitation wavelengths.

HT from photoexcited m-ITIC (at 700 nm) is only one of the channels for current generation in polymer:NFA heterojuctions, so that we also examine polymer excitation at 480 nm. We compare in Fig. 2a (top) the TA dynamics for the optimized J61:m-ITIC (1:1) BHJ with both excitation wavelengths. Only in the case of 480 nm excitation, charges appear at the shortest measurable time that is defined by the 60 fs time resolution of our experiment. Detailed analysis of the TA spectrum at 0.1 ps reveals that about 36% J61 excitons, 28% m-ITIC excitons and 36% charges are present (Supplementary Table 3), while only m-ITIC excitons are observed at the early time delay with selective acceptor excitation at 700 nm (Supplementary Fig. 7a). We use kinetic modeling of the TA dynamics to elucidate the underlying photophysics (Fig. 2a (bottom), Supplementary Note 4). Multiphasic and diffusion-mediated processes are approximated by average time constants, causing small discrepancies with the experimental data. For 480 nm excitation, three phenomena are observed (Fig. 2b, c): (i) about 28% of absorbed photons lead to direct excitation of m-ITIC (in agreement with the absorption spectrum, Supplementary Fig. 7b), which then undergoes intrinsic and diffusion-mediated HT similar as is modeled with 700 nm excitation. (ii) About half of all J61 excitons are generated close to a m-ITIC interface and undergo ET within the time resolution of the experiment. This leads to ultrafast appearance of charges in shorter than 60 fs, which is not observed for HT with m-ITIC excitation. (iii) The other J61 excitons, generated further away from an interface in the ordered J61 domains, undergo diffusion-mediated ET in competition with excitation energy transfer (EET), whereby EET takes over due to a shallower distance dependence compared to ET. The absorption spectrum of m-ITIC overlaps indeed with the emission spectrum of J61 (Supplementary Fig. 2), and we calculate a Förster radius of 5.2 nm (see Supplementary Note 5), in agreement with other NFA systems^50,51^. EET populates m-ITIC excitons near an interface, which then undergo HT with the intrinsic 0.8 ps time constant. As the population of m-ITIC excitons is gradually replenished by EET from J61, the faster m-ITIC exciton decay obtained at 700 nm is not observed at 480 nm. We conclude that intrinsic ET is ultrafast (shorter than 60 fs) and that delayed charge generation with 480 nm excitation occurs predominantly via HT following direct m-ITIC excitation or EET to m-ITIC. Overall, our results demonstrate an unbalance of the ET and HT rates in the same J61:m-ITIC (1:1) blend, with HT being over an order of magnitude slower (intrinsic time constant of 0.8 ps vs. <60 fs for ET).

Given the clean interface and well-defined excitation profiles obtained by transfer-matrix modeling (TMM, Supplementary Note 6) in the J61:m-ITIC bilayer, this sample is used to confirm the above findings and to evaluate the distance over which J61 and m-ITIC excitons can undergo intrinsic CT without need for diffusion. With 480 nm excitation, we predict from the excitation profile that 77% of all absorbed photons are absorbed by J61 and 23% by m-ITIC (Fig. 2d). This corresponds well to the sum of J61 excitons and charges (59 + 16% = 75%) and to the m-ITIC exciton population (25%) in the early 0.1 ps TA spectrum, respectively (Supplementary Fig. 7a, Supplementary Table 3). We therefore confirm that the ultrafast charge generation in J61:m-ITIC with 480 nm excitation is due to prompt ET from photoexcited J61 and that some m-ITIC is directly excited. In the bilayer, we find a percentage of excitons undergoing quenching by intrinsic HT (13% in 0.9 ps with 700 nm excitation, Supplementary Table 1) and by intrinsic ET (16% in <60 fs with 480 nm excitation, Supplementary Table 3) corresponding to excitons generated within 1.2 and 1.9 nm from the interface for the acceptor and donor, respectively (as calculated from the excitation profiles in Fig. 2d). This shows that J61 excitons generated slightly further away from an interface can undergo ET without needing to diffuse, likely due to a higher exciton delocalization in the conjugated polymer compared to the small molecule^52^. The same is observed in the J61:m-ITIC (1:1) blend, where we find 34% charge rise due to intrinsic ET (480 nm), but only 14% due to intrinsic HT (700 nm, Table 1). Together with different J61 and m-ITIC domain sizes, leading to different bulk-to-interface ratios, the exciton delocalization can explain the higher weight of intrinsic ET in the BHJ sample.

### Driving force dependence in different polymer:m-ITIC blends

**Fig. 3:**
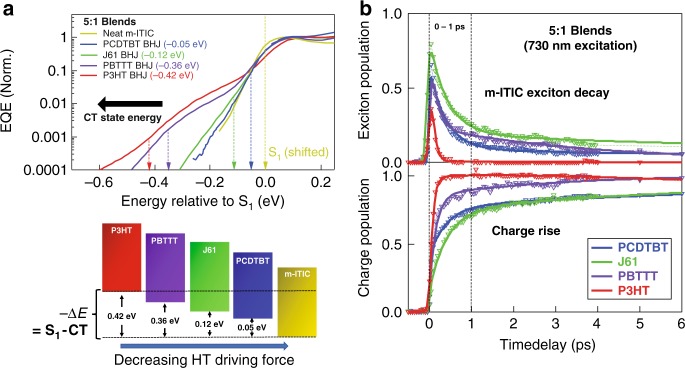
Driving force dependent sub-picosecond HT in polymer:m-ITIC blends. **a** sEQE spectra for the polymer:m-ITIC 5:1 blends and neat m-ITIC. The curves are shifted to always have the m-ITIC S_1_ energy (from a bi-Gaussian fit of the sEQE and EL data) at 0 eV for better comparison. At the bottom, a schematic illustration of the driving forces for HT in polymer:m-ITIC 5:1 BHJ samples is shown. **b** m-ITIC exciton decay (top) and charge rise (bottom) dynamics for polymer:m-ITIC 5:1 BHJ samples, upon selective m-ITIC excitation at 730 nm, obtained from the analysis of the TA data. The *y*-axis is expressed as a fraction of the total absorbed photon density. Symbols are the experimental data and solid lines are exponential fits obtained globally for the exciton decay and charge rise.

Having unambiguously established a sub-picosecond intrinsic HT rate in the J61:m-ITIC sample with only 0.12 eV driving force, we now further address the question of how this driving force affects the HT rate in different polymer:NFA blends with varied energetics. To this effect, we continue the strategy to dilute the m-ITIC acceptor in different polymer matrices (5:1 blends). These polymers exhibit similar absorption spectra as J61 (allowing for selective m-ITIC excitation, Supplementary Fig. 2), but different CT energy levels and thus HT driving forces when combined with m-ITIC (Fig. 3a). This is clearly depicted in the sEQE spectra by the gradual shift of the CT band in the sub-bandgap region with respect to the acceptor S_1_ state (see complete sEQE/EL analysis in Supplementary Fig. 8 and Supplementary Table 2). When we trace the decay of m-ITIC excitons and the concomitant rise of the charges obtained from the analysis of the TA data, we find that all 5:1 blends undergo significant HT already within 1 ps (Fig. 3b). The fastest time constant (intrinsic HT without exciton diffusion) always has a significant weight (larger than 60%) due to effective m-ITIC dispersion (Table 1). Importantly, this time constant remains on the sub-picosecond time scale (0.4 ps) even when the driving force approaches zero (0.05 eV). HT then gets faster with increasing driving force and becomes ultrafast (0.08 ps) above 0.4 eV. To access also the intrinsic ET rates at different energetics, TA measurements with 480 nm excitation on 1:1 polymer:m-ITIC blends were carried out and reveal an ultrafast (shorter than 60 fs) ET component for all donors (Supplementary Fig. 9, Table 1).

**Fig. 4:**
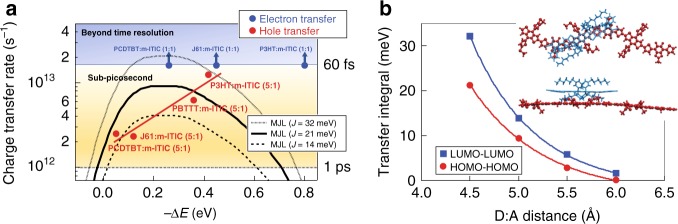
Relationship between the driving force and the HT and ET rates. **a** The charge-transfer rate (inverse of the first time constant obtained from the analysis of the TA dynamics) for ET and HT in different polymer:m-ITIC BHJ blends is plotted against the driving force (obtained from the sEQE and EL spectra). The red solid line is a guide for the eye to show the trend in HT rate, while the black curves represents the CT rate predicted from semiclassical Marcus–Levich–Jortner theory (with transfer integral *J*_DA_ = 14– 32 meV, Huang−Rhys parameter *S* *=* 1 and outer re-organization energy *λ*_ο_ = 0.15 eV). **b** LUMO–LUMO transfer integral for ET and HOMO–HOMO transfer integral for HT in a J61:m-ITIC complex as a function of the donor:acceptor (D:A) distance. The geometry as obtained by DFT calculations (at 4.5 Å) is depicted in the inset.

We summarize our findings about the driving force dependence of HT and ET in polymer:m-ITIC systems in Fig. 4a, where we plot the intrinsic CT rate (inverse of the first time constant obtained from the analysis of the TA dynamics) against its corresponding driving force. Note that using the average charge rise time instead does not reveal clear trends (Supplementary Fig. 10), due to the random contributions of exciton diffusion, non-optimal D:A conformations and EET (at 480 nm). Within the time resolution of our experiment, the intrinsic ET rates remain ultrafast (shorter than 60 fs) in the 0.3–0.8 eV driving force range, similar to what has been reported in typical polymer:fullerene blends^10^. On the other hand, there is a clear dependence of the intrinsic HT rate on the energetics, with CT times varying on the sub-picosecond scale (from 0.5 to 0.08 ps) for driving forces from 0.05 to 0.4 eV. Such fast rates are crucial to be competitive with the natural m-ITIC exciton lifetime during HT. Although the exciton lifetime of m-ITIC (and ITIC derivatives in general) is highly multiphasic and dependent on the environment and molecular packing (see dynamics in neat m-ITIC film, m-ITIC solution and m-ITIC:polystyrene blends in Supplementary Fig. 11)^16,53^, we find that the fastest component decays with a 3 ps time constant for m-ITIC molecules in the neat film. In the 1:1 blend, the X-ray data indicates the presence of m-ITIC-rich domains, where we expect a comparable packing and short exciton lifetime. Therefore, for HT to be efficient, the observed few-hundred femtosecond CT times at low driving force are essential to prevent any exciton loss mechanisms.

## Discussion

To evaluate whether the observed CT rates are consistent with a Marcus type description, we have carried out DFT calculations of the transfer integral (electronic coupling) for the ET and HT processes in complexes of J61 and m-ITIC, which is the highest-efficiency OPV system of our study. We find an almost co-facial structural fit of the acceptor molecule on top of the donor polymer that maximizes the overlap of the molecular cores despite sterically demanding side chains (Fig. 4b, Supplementary Note 7). The transfer integral is higher (32 meV) for ET (LUMO–LUMO) than for HT (21 meV, HOMO–HOMO), which might be at the origin of the generally higher ET rates compared to HT rates in the investigated polymer:m-ITIC systems, even at similar driving force. The electronic coupling dramatically decreases when the D:A distance increases beyond the distance of 4.5 Å (Fig. 4c), highlighting that slower CT rates can occur even without exciton diffusion for non-optimal geometries in the blend. Furthermore, we simulated the individual molecular relaxation energies and we find a very low re-organization energy of 161 meV for the J61:m-ITIC CT complex (105 and 56 meV for m-ITIC anion and J61 cation, respectively). With the calculated values, we predict an intrinsic HT time of 0.13 ps using the semiclassical Marcus–Levich–Jortner (MLJ) model for a driving force of 0.12 eV (Supplementary Note 8)^5,33,54^. This is close to the experimental sub-picosecond value of 0.4 ps, which is thus consistent with a MLJ description in the Marcus normal region, at moderate electronic coupling (21 meV) and at low re-organization energy (161 meV). Indeed, the Marcus formalism predicts a maximal CT rate when the driving force and re-organization energy are similar, so that a low re-organization energy shifts the low driving force region towards higher rates. An ET time of 0.08 ps is predicted for a driving force of 0.45 eV (Marcus inverted region) and a relatively high transfer integral of 32 meV, which in principle also agrees with the ultrafast experimental value (shorter than 0.06 ps).

Although the measured trend of increasing HT rate with driving force agrees qualitatively with the MLJ model in the normal region (Fig. 4a), one should keep in mind that the re-organization energy and transfer integrals of the other donor:m-ITIC systems can be different (MLJ curves for different electronic couplings are included in Fig. 4a to illustrate the effect on the CT rates). We also note that the inverted region (decrease in rate upon increase in driving force) is experimentally not observed for the HT and ET rates, which can be mainly assigned to the broadness of the MLJ rate spectrum^55^. The absence of the Marcus inverted region for polymer:fullerene blends has also been attributed to the fact that sub-100 fs rates are too fast to be described within the Marcus formalism for non-adiabatic ET, warranting a description in the adiabatic limit^30–33^. It is therefore likely that MLJ is not the best model to describe the ultrafast ET rates in our NFA systems, even if the predicted value matches the measured rate for m-ITIC:J61. Finally, strong coupling and a low driving force (CT state close to S_1_ state) can lead to hybridized locally excited (LE) and CT states^8,56^. However, given the moderate electronic coupling (21 meV) compared to a driving force of 120 meV, hybridization should not contribute more than 3% to the HT mechanism in J61:m-ITIC (although we cannot exclude more hybridization in the near-zero driving force PCDTBT:m-ITIC complex). Negligible hybridization is consistent with the distinct CT and S_1_ features in the sEQE/EL spectra (for J61, pBTTT and P3HT blends), the absence of hybridized state signatures in the TA^57^, and the clean conversion of the S_1_ excitons (with similar spectral signatures as in neat m-ITIC) to charges (which do not change spectral shape in time).

In conclusion, we have investigated here both the electron and hole transfer dynamics in heterojunctions of the non-fullerene acceptor m-ITIC with four polymeric donors having different driving forces and sample configurations. In contrast to previous work, we have decoupled the effects of morphology-dependent exciton diffusion from the intrinsic CT rates, by comparing both optimal and dilute bulk heterojunctions with bilayer samples. We demonstrate an unbalance of the electron and hole transfer processes in the high-efficiency J61:m-ITIC system (hole transfer is slower), due to different transfer integrals and driving forces for the two pathways. In contrast, both the electron and hole transfer rates are ultrafast in typical polymer:fullerene systems^32^, sparking future interest in the efficiency of charge generation in NFA blends in regions of donor and acceptor excitation. Our main conclusion is that in spite of this unbalance, the intrinsic hole transfer is much faster than in earlier reports and remains on the sub-picosecond time scale in all investigated samples, even for a near-zero driving force. Sub-picosecond hole transfer rates at low driving force are consistent with the predictions in the Marcus normal region for CT with moderate electronic coupling, whereby the high rates can be explained by a small reorganization energy. Hybridization of the excited and CT state is negligible for our highest-efficiency J61:m-ITIC system, which is possibly advantageous in terms of reducing charge recombination^56^. Overall, we show that the driving force for interfacial CT does not present a limiting factor for efficient CT at near zero energy loss, as long as the morphology and donor–acceptor geometry are optimized.

## Methods

### Sample preparation

Poly[[9-(1-octylnonyl)-9H-carbazole-2,7-diyl]-2,5-thiophenediyl-2,1,3-benzothiadiazole-4,7-diyl-2,5-thiophenediyl] (PCDTBT) was purchased from Brilliant Matters, poly(3-hexylthiophene) (P3HT) and poly[2,5-bis(3-tetradecylthiophen-2-yl)thieno[3,2-b]thiophene] (PBTTT) were purchased from Sigma-Aldrich, while 3,9-bis(2-methylene-((3-(1,1-dicyanomethylene)-6/7-methyl)-indanone))-5,5,11,11-tetrakis(4-hexylphenyl)-dithieno[2,3-d:2′,3′-d′]-s-indaceno[1,2-b:5,6-b′]dithiophene (m-ITIC) and poly[[5,6-difluoro-2-(2-hexyldecyl)-2*H*-benzotriazole-4,7-diyl]-2,5-thiophenediyl[4,8-bis[5-(dodecylthio)-2-thienyl]benzo[1,2-*b*:4,5*b*′]dithiophene-2,6-diyl]-2,5-thiophenediyl] (J61) were synthesized following the method previously reported^34^. Note that certain commercial equipment, instruments, or materials are identified in this paper in order to specify the experimental procedure adequately. Such identification is not intended to imply recommendation or endorsement by the National Institute of Standards and Technology (NIST), nor is it intended to imply that the materials or equipment identified are necessarily the best available for the purpose.

For the TA experiments, neat m-ITIC films were deposited by spin-coating m-ITIC solution in chloroform (CF) onto quartz substrates at 157 rad s^−1^ (1500 rpm) for 1 min, resulting in film a thickness of 9 nm (1.25 mg mL^−1^), as measured by a Dektak depth profilometer. Neat J61 films were prepared in a similar manner, spin-coated by using CF with a concentration of 1.25 mg mL^−1^. For bilayers, we prepared a poly(sodium 4-styrenesulfonate) (PSS) layer by spin-coating PSS solution in water onto a glass substrate, and subsequently spin-coated J61 onto the PSS. Next, the glass:PSS:J61 sample was placed upside down onto a m-ITIC-coated quartz substrate. A drop of water placed on the edge of these two substrates dissolved the PSS, resulting in the transfer of the J61 layer onto the m-ITIC layer (see Supplementary Fig. 12). The bulk heterojunctions were prepared by mixing the polymer and m-ITIC with either 1:1 or 5:1 mass ratio (with polymer concentration of 7.5 mg mL^−1^) in CF, and spin-coating this solution at 3500 rpm for 1 min. The absorption spectra were recorded with a PerkinElmer Lambda 950 spectrophotometer. For structural characterization by x-ray diffraction, the films were deposited on Si (100) substrates. The solar cells for sEQE and EL experiments were fabricated by using an ITO/ZnO/Active layer/MoO_3_/Ag architecture. ZnO was prepared by mixing zinc acetate and ethanolamine in 2-methoxyethanol, and then spin-coating onto pre-cleaned ITO substrates (ultra-sonicated in detergent, water, acetone and isopropanol), after which annealing at 150 °C was carried out for 30 min. The active layer was spin-coated in the same manner as the films prepared for the TA samples. MoO_3_ and Ag were then thermally evaporated as electrodes.

### sEQE photovoltaic measurements

For the sEQE measurements, the light of a quartz halogen lamp (50 W), chopped at 140 Hz, was coupled into a monochromator (Newport Cornerstone 260 1/4m, USA). The resulting monochromatic light was focused onto the organic solar cell, its current at short-circuit conditions was fed to a current pre-amplifier (DHPCA-100, FEMTO Messtechnik GmbH, Germany) before it was analyzed with a lock-in amplifier (Signal Recovery 7280 DSP, USA). The time constant of the lock-in amplifier was chosen to be 0.5 s and the amplification of the pre-amplifier was increased to resolve low photocurrents. The EQE PVs is determined by dividing the photocurrent of the OPVs by the flux of incoming photons, which was obtained with a calibrated silicon (Si) and indium–gallium–arsenide (InGaAs) photodiode.

### EL measurements

The EL measurements were performed using an Andor SR-303i-B spectrometer equipped with a silicon (Si) (DU420A-BR-DD) and an indium–gallium–arsenide (InGaAs) (DU491A-1.7) detector. Voltage was supplied by a Keithley 2400 source meter and was typically 1 V. For some low signal devices, the voltage was raised up to 3 V in order to get enough EL response. To prevent device heating when the EL signal was measured in the near-IR, the voltage was pulsed for 90 s. The voltage used was the same throughout the entire spectrum (for the silicon and germanium detectors) and then the final continuous EL spectrum was obtained, after subtraction of the dark background signal.

### TA spectroscopy

TA experiments were carried out using a home-built femtosecond pulsed pump-probe laser setup. The excitation pump pulses at 480, 700, or 730 nm were generated with a commercial optical parametric amplifier (OPerA Solo, Coherent) from the fundamental 800 nm laser output from a Ti:sapphire laser system with regenerative amplification, providing 35 fs pulses at a repetition rate of 1 kHz (Astrella, Coherent). These pump wavelengths were chosen to selectively excite the donor polymers or the acceptor. 730 nm excitation was used in the 5:1 blends to exclude direct donor excitation due to its high loading. The pump energy at the sample position was adjusted to be in a linear regime where the dynamics were independent of fluence, without any bimolecular recombination effects, which corresponds to a low fluence in the range of 1 μJ cm^−2^ with a pump beam diameter around 1.2 mm (determined with a BC106-Vis Thorlabs beam profiler, 1:e^2^ cut-off) (see Supplementary Fig. 13). Moreover, the fluence was corrected to have a similar flux of absorbed photons (3 × 10^11^ photons cm^−2^) for all the measurements, taking into account the absorbance at the excitation wavelength and the photon energy. The probe beam consisted of a white light continuum (500–780 nm, visible range and 800–1220 nm near-IR range) generated by passing a portion of the 800 nm amplified Ti:sapphire output through a 5 mm-thick sapphire window. Either a 720 nm low pass or a 850 nm high pass filter was used to remove the remaining fundamental intensity from the white light. The visible and the near-IR parts of the spectrum were recorded separately. The probe intensity was negligible compared to the pump intensity and the spot size was much smaller (probe energy of lower than 5 nJ, probe diameter of about 200 μm). The probe pulses were time delayed with respect to the pump pulses by means of a computer-controlled translation stage in order to record the dynamic traces. The probe beam was split before the sample into a signal beam (transmitted through the sample and overlapped with the pump beam) and a reference beam. The signal and reference beams were detected separately using a pair of spectrographs (home-built prism spectrometers) equipped with 512 × 58 pixels back-thinned Silicon CCDs (Hamamatsu S07030-0906) and InGaAs arrays (Hamamatsu) for, respectively, visible and near-IR detection. The spectrographs were assembled by Entwicklungsbüro Stresing, Berlin. Wavelength calibration was accomplished with a set of 10 nm bandpass filters. To improve the sensitivity, the pump pulses were chopped at half the amplifier frequency, and the transmitted intensity of the signal beam was recorded shot-by-shot and it was, finally, corrected for laser intensity fluctuations using the reference beam. The spectra were averaged 3000–4500 times at each time delay and the entire range of measured time delays was scanned 8–10 times. All the TA experiments were performed with a probe polarization at the magic angle with respect to the one of the pump in order to avoid effects of the polarization of the excitation beam on the probed absorption intensity. Prior to the TA analysis, the spectra were corrected for the chirp of the white light (parameters obtained by measuring the pump-probe cross-correlation on a glass slide placed between crossed polarizers).

### X-ray scattering: GIWAXS and R-SoXS

GIWAXS measurements were performed at the 11-BM Complex Materials Scattering (CMS) beamline of the National Synchrotron Light Source II (NSLS-II) with a beam energy of 10 keV. The 2D scattering patterns were collected at an X-ray incidence angle of 0.12° with a Pilatus 800K detector with a pixel size of 101.7 μm and placed about 230 mm from the sample. The data were analyzed using Nika^58^ analysis package based on Igor Pro. Sector averaged 1D scattering profiles were obtained from 15° cake sectors. Volume normalized pole figures were constructed from the 2D GIWAXS images corrected for the missing wedge by integrating the intensities at each detector azimuth within the *q* range of the lamellar diffraction peak. A linear background defined by the intensities at the two ends of the integrated *q* range was subtracted. The relative degree of crystallinity (rDoC) was calculated by integrating the volume normalized intensities over the crystallographic orientation sphere: ${\mathrm{rDoC}} = {\int}_0^{\pi /2} {I(\chi )\sin \chi \,{\mathrm{{d}}}\chi }$. Film thicknesses were measured using an ellipsometer (M-2000-XI, J. A. Woollam Co., Inc). Spacing and coherence lengths for the J61 and m-ITIC were calculated from the (100) peaks by fitting Gaussian peaks to the 1D sector-averaged profiles (see Supplementary Table S4). Coherence lengths were calculated using the Scherrer equation^59^.

For R-SoXS, the scattering contrast is directly proportional to Δ*n*^2^ = Δ*δ*^2^ + Δ*β*^2^, where 1−*δ* is the real part (related to dispersion) and *β* is the imaginary part (related to absorption) of the complex refractive index. R-SoXS measurements were performed in transmission geometry at the ALS beamline 11.0.1.2^60^ following procedures described earlier^61^. BHJ films were cast on PSS-coated glass substrates, floated in water, and transferred onto 100 nm Si_3_N_4_ windows (Norcada)^62^. The 2-D R-SoXS data were collected at beamline 11.0.1.2 at the Advanced Light Source using a Peltier cooled (−45 °C) in-vacuum (base pressure ≈ 10^−9^ kPa (10^−8^ mBar)) CCD detector (PI-MTE, Princeton Instruments, 2048 × 2048 pixels). 1-D scattering profiles were obtained from the reduction of the 2-D scattering patterns using a custom Nika analysis package and subsequently normalized for the instantaneous X-ray flux. The scattering intensity is affected by the distance traveled by the X-ray beam through the sample as well as the scattering volume. R-SoXS scattering intensities were therefore normalized for absorption and film thickness. Data were acquired at multiple energies in the range 283–284 eV to optimize material contrast over the mass–thickness contrast, minimize beam damage, and avoid fluorescence background^63,64^. Material contrast at the C K-edge was calculated from transmission NEXAFS measured using a photodiode at the ALS beamline 6.3.2 on neat films transferred onto 100 nm Si_3_N_4_ windows (Norcada). The NEXAFS spectra were normalized to the absorption spectrum of an identical blank Si_3_N_4_ window. The spectra were analyzed using the QANT analysis package^65^.

### Spectroscopic ellipsometry

The real (*n*) and imaginary (*k*) parts of the complex optical constants were determined from variable angle spectroscopic ellipsometry (VASE) measurements on neat polymer (J61) and acceptor (m-ITIC) films cast on Si (see results in Supplementary Table 5). VASE was carried out with a M-2000-XI, J.A. Woollam Co., Inc., USA. The incidence angle was scanned from 45° to 75° in steps of 15°, and the photon energy varied from 0.74 to 5.87 eV. Modeling of the blend films was performed using the CompleteEASE software package. The film thickness and optical constants were fitted to a uniaxial B-Spline model. The ordinary values of the *n* and *k* are reported here.

### DFT calculations

In order to calculate the electron transfer parameters *λ*_i_ and ***J***_DA_, we performed density functional theory calculations as implemented in Gaussian 09^66^. In particular, we used the B3LYP^67,68^ exchange-correlation functional and the 6-21G basis set for optimizing the molecular structures of monomers and donor-acceptor complexes and for determining the total energy. We calculated the transfer integral for which we used the larger 6-311G** basis for a better description of the electronic states. In order to obtain the relaxation energies, we firstly optimized the molecular structures of neutral donors and acceptors individually (see Supplementary Note 7), to obtain their equilibrium geometry (short-hand notation ***R***_0_) and the related total energies *E*(***R***_0_). As displayed in Supplementary Figs. 18 and 19, both the m-ITIC central unit and the J61 polymer backbone are rather planar, i.e. torsion angles are below 5°. The attached phenyl rings (for m-ITIC) and thiophene rings (for J61), however, are pointing out of the backbone plane. These groups therefore increase the molecular distance when forming *π*–*π* stacks. Secondly, the negatively charged m-ITIC and the positively charged J61 structures were optimized, which results in the geometries of the ionic species (***R***_±_ respectively). Finally, a single point calculation of the uncharged molecules in the geometries ***R***_±_ were performed to get the total energy *E*(***R***_±_). The relaxation energies were then obtained from the differences ***λ***_0→±_ = *E*(***R***_±_)−*E*(***R***_0_) for J61 and m-ITIC, respectively. The results are summarized in Supplementary Table 6. Note, that the different conformations of the polymer yield almost the same values for ***λ***_0→+_. This led us to the conclusion that the relaxation energy is mainly determined by local interaction of nearest and next-nearest atoms and is therefore independent from the global orientation of the backbone. Moreover, Supplementary Table 6 shows that turning one thiophene ring in each repeat unit from *cis* to *trans* orientation leads to an increase of about 126 meV in the total energy for the structure in Supplementary Fig. 19. Hence, the *cis*/*cis* orientation exhibits the smallest energy such that only this conformation is considered in the following. The relaxation energies, which are assigned mainly to intramolecular high-frequency modes due to the stiff backbone, of both donor and acceptor add to the intramolecular reorganization energy *λ*_i_ of the CT process. For the polymer, we take the relaxation energy for the structure consisting of two repeat units as this is similar in size to the acceptor molecule, hence *λ*_*i*_ = *λ*_0→+_ + *λ*_0→−_ = 161 meV.

For the simulation of donor–acceptor complexes to calculate the transfer integrals, the obtained molecular structures for m-ITIC and J61 were used to construct geometries with different lateral and vertical distances. We considered intermolecular distances ranging from 4.5 to 6.0 Å (see Supplementary Note 7), the results of which are depicted and discussed in the main text.

## Supplementary information

Supplementary Information

## Data Availability

Data in Figs. 1–4 is made publicly available (BORIS Repository, University of Bern, 10.7892/boris.139648).
