# Supplementary material for: Sub-picosecond charge-transfer at near-zero driving force in polymer:non-fullerene acceptor blends and bilayers
Source: Nat Commun. 2020 Feb 11;11:833. doi: 10.1038/s41467-020-14549-w (PMC7012859; doi:10.1038/s41467-020-14549-w)
Supplement: Supplementary file 1 — Supplementary Information [file 41467_2020_14549_MOESM1_ESM.pdf]

Supplementary Information

Sub-Picosecond Charge-Transfer at Near-Zero Driving Force in Polymer:Non-Fullerene Acceptor Blends and Bilayers

Yufei Zhong,[1†] Martina Causa',[1†] Gareth John Moore,[1] Philipp Krauspe,[1] Bo Xiao,[2] Florian Günther,[3] Jonas Kublitski,[4] Rishi Shivhare,[4] Johannes Benduhn,[4] Eyal BarOr,[5] Subhrangsu Mukherjee,[6] Kaila M. Yallum,[1] Julien Réhault,[1] Stefan C. B. Mannsfeld,[4] Dieter Neher,[5] Lee J. Richter,[6] Dean M. DeLongchamp,[6] Frank Ortmann,[7] Koen Vandewal,[8] Erjun Zhou,[2]* Natalie Banerji[1]*

† These authors contributed equally

[1]Department of Chemistry and Biochemistry, University of Bern, Freiestrasse 3 CH-3012 Bern, Switzerland. Email: natalie.banerji@dcb.unibe.ch

[2]Chinese Academy of Sciences (CAS) Key Laboratory of Nanosystem and Hierarchical Fabrication, CAS Center for Excellence in Nanoscience, National Center for Nanoscience and Technology, Beijing 100190, P. R. China. E-mail: zhouej@nanoctr.cn

[3]Instituto de Física de São Carlos (IFSC), Universidade de São Paulo (USP), Av. Trabalhador saocarlense, 400, CEP 13560-970 São Carlos, Brazil

[4]Dresden Integrated Center for Applied Physics and Photonic Materials (IAPP) and Institute for Applied Physics Technische Universität Dresden, Nöthnitzer Str. 61, 01187 Dresden, Germany

[5]Institute of Physics and Astronomy, University of Potsdam, Karl-Liebknecht-Str. 24-25, 14476 Potsdam-Golm, Germany

[6]Material Measurement Laboratory, National Institute of Standards and Technology (NIST), Gaithersburg, Maryland 20899, USA

[7]Center for Advancing Electronics Dresden, Technische Universität Dresden, Helmholtzstr. 18, 01062 Dresden, Germany

[8]Institute for Materials Research (IMO-IMOMEC), Hasselt University, Wetenschapspark 1, 3590 Diepenbeek, Belgium

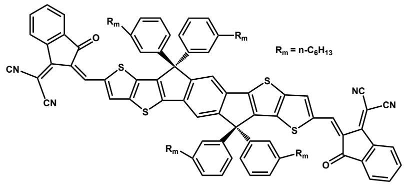

m-ITIC

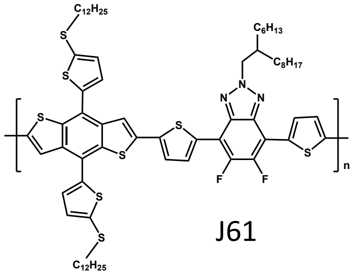

J61

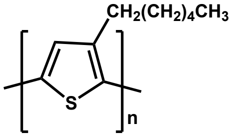

P3HT

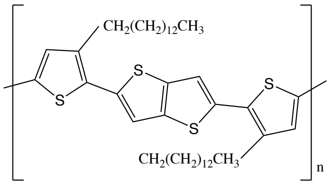

PBTTT

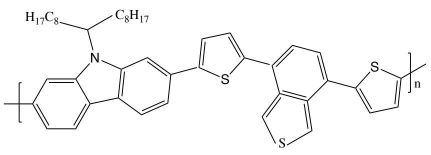

PCDTBT

Supplementary Figure 1. **Chemical structures of materials**. Chemical structure of m-ITIC, J61, P3HT, PBTTT and PCDTBT.

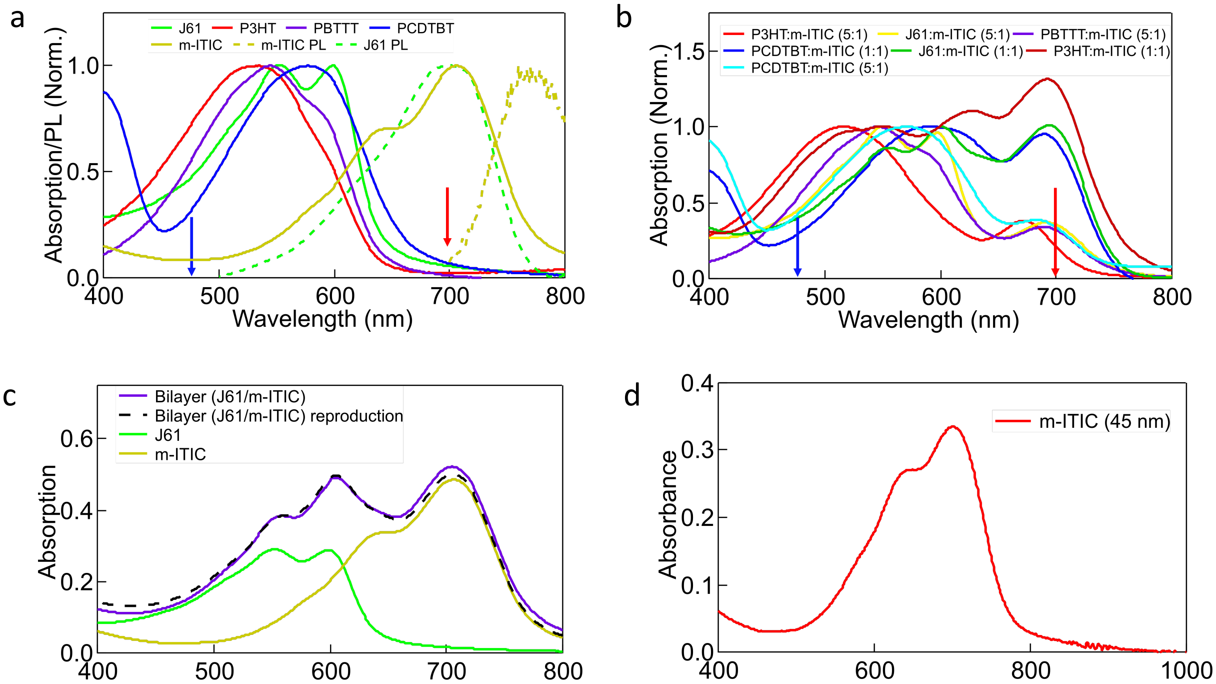

§

Supplementary Figure 2. **Steady-state absorption spectra**. **a**. Normalized absorption spectra of the neat m-ITIC and polymer films, and photoluminescence (PL) spectra of m-ITIC and J61. **b**. Absorption spectra of the different polymer:m-ITIC blends investigated here (normalized at the polymer absorption peak). The arrows indicate excitation wavelengths used in TA (blue: 480 nm, red: 700 nm). **c**. Absorption spectrum of the J61:m-ITIC bilayer and neat J61 and m-ITIC components. The absorption of bilayer can be reproduced by the sum of absorption spectrum of J61 and m-ITIC. **d**. Absorbance of m-ITIC film measured in an integrating sphere.

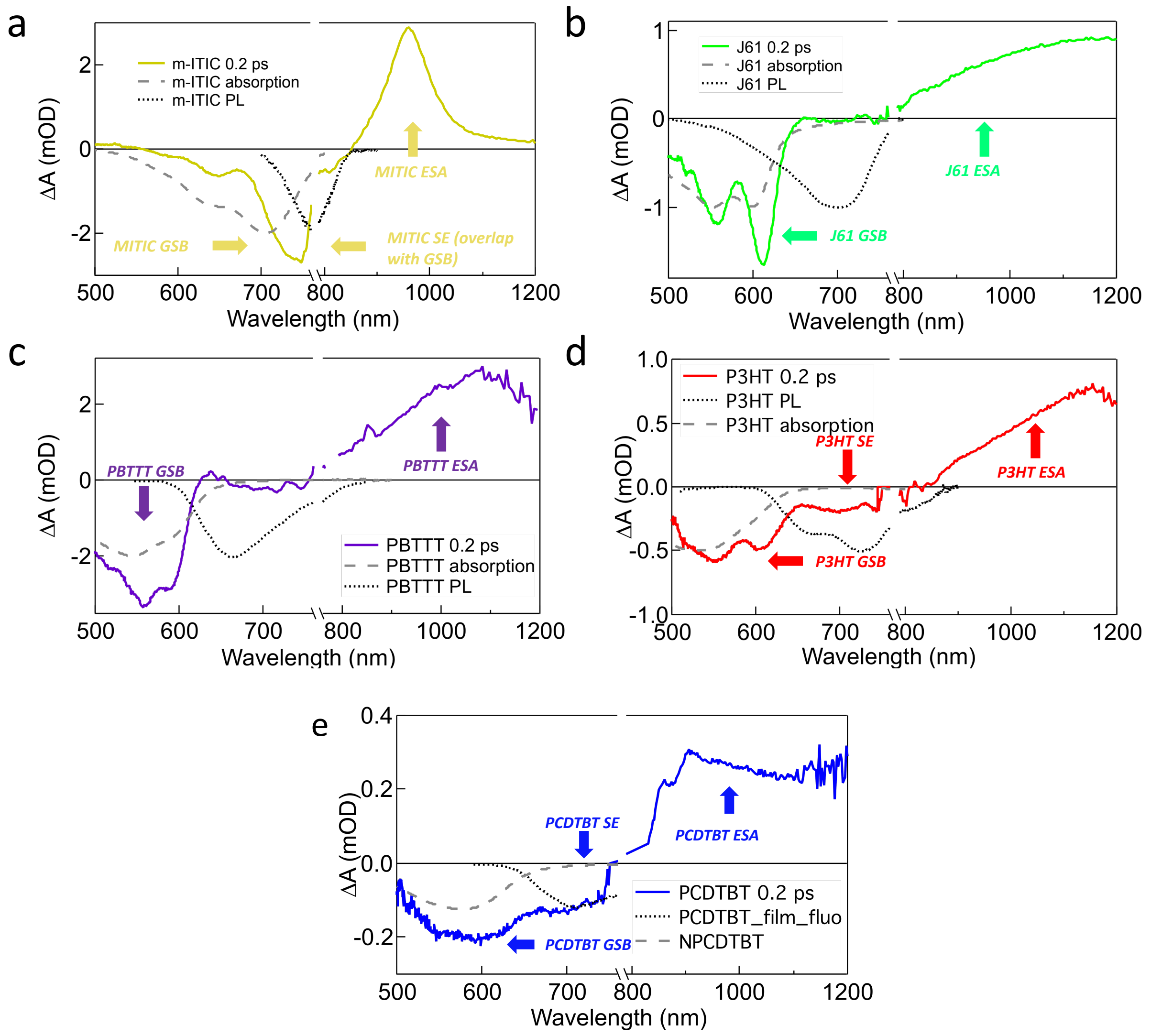

Supplementary Figure 3. TA spectra of the neat polymers and m-ITIC. Early TA spectra of the neat films of the investigated materials, showing the signatures of the excitons, including the ground state bleaching (GSB), the stimulated emission (SE) and the excited state absorption (ESA). **a.** m-ITIC, **b.** J61, **c.** PBTTT, **d.** P3HT and **e.** PCDTBT. The m-ITIC film shows a negative peak at 730 nm, which is a combination of the GSB and SE, as seen from the absorption and PL spectra. In the near-IR region, the peak at 960 nm represents the ESA. For the polymers, the GSB in the visible region mirrors their absorption spectrum. SE of P3HT and PCDTBT appears around 700 nm. All polymers show broad ESA bands in the near-IR region. The (negative) absorption and emission spectra of the films are shown as well. Their intercept Is taken as the S_1 energy.

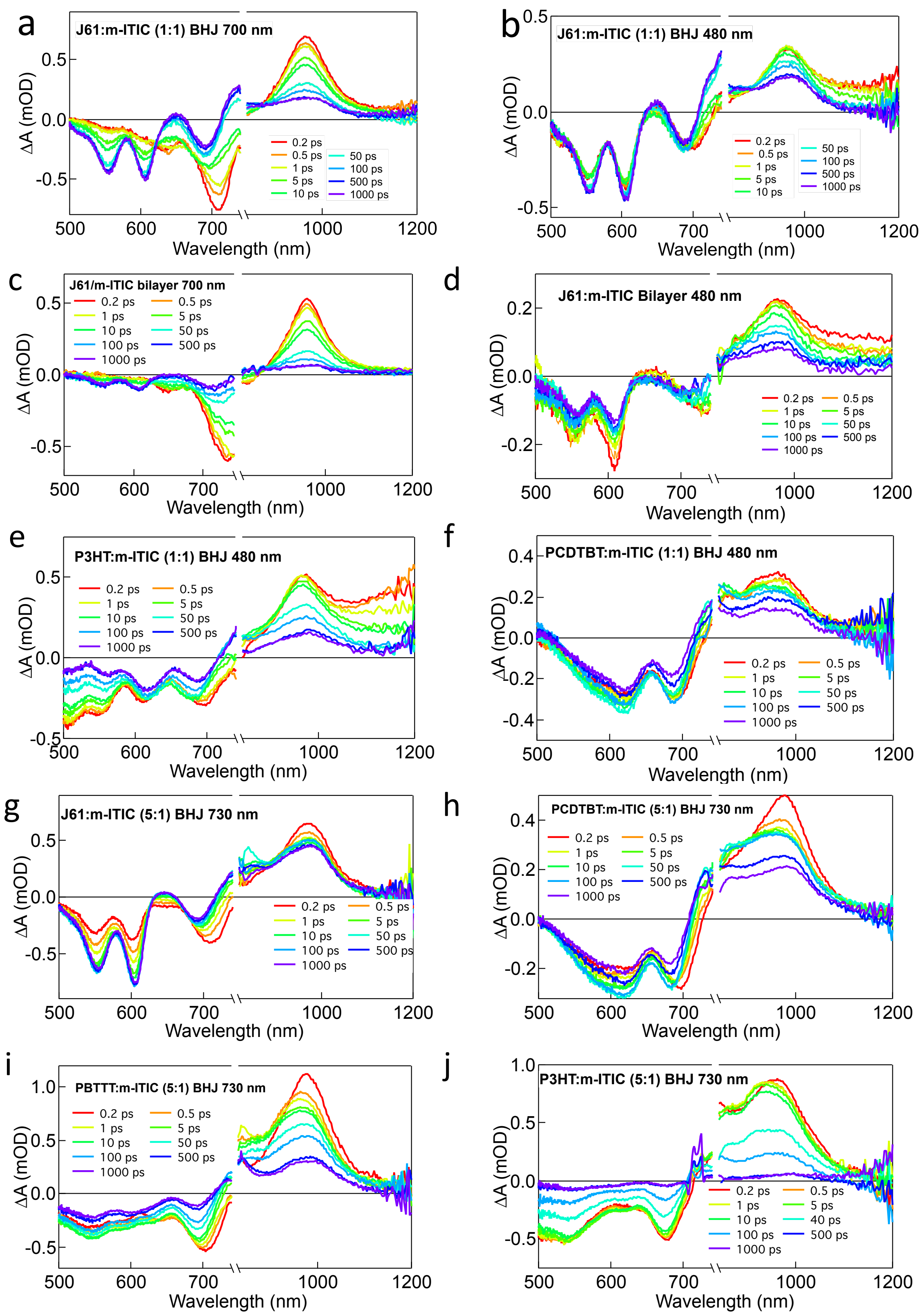

Supplementary Figure 4. **TA spectra of the polymer:m-ITIC BHJ blends and bilayers.** TA spectra at selected time delays for the investigated blends and bilayers at different excitation wavelengths. Note that the signature of the charge absorption coincides with the m-ITIC ESA (at 960 nm), but with lower oscillator strength.

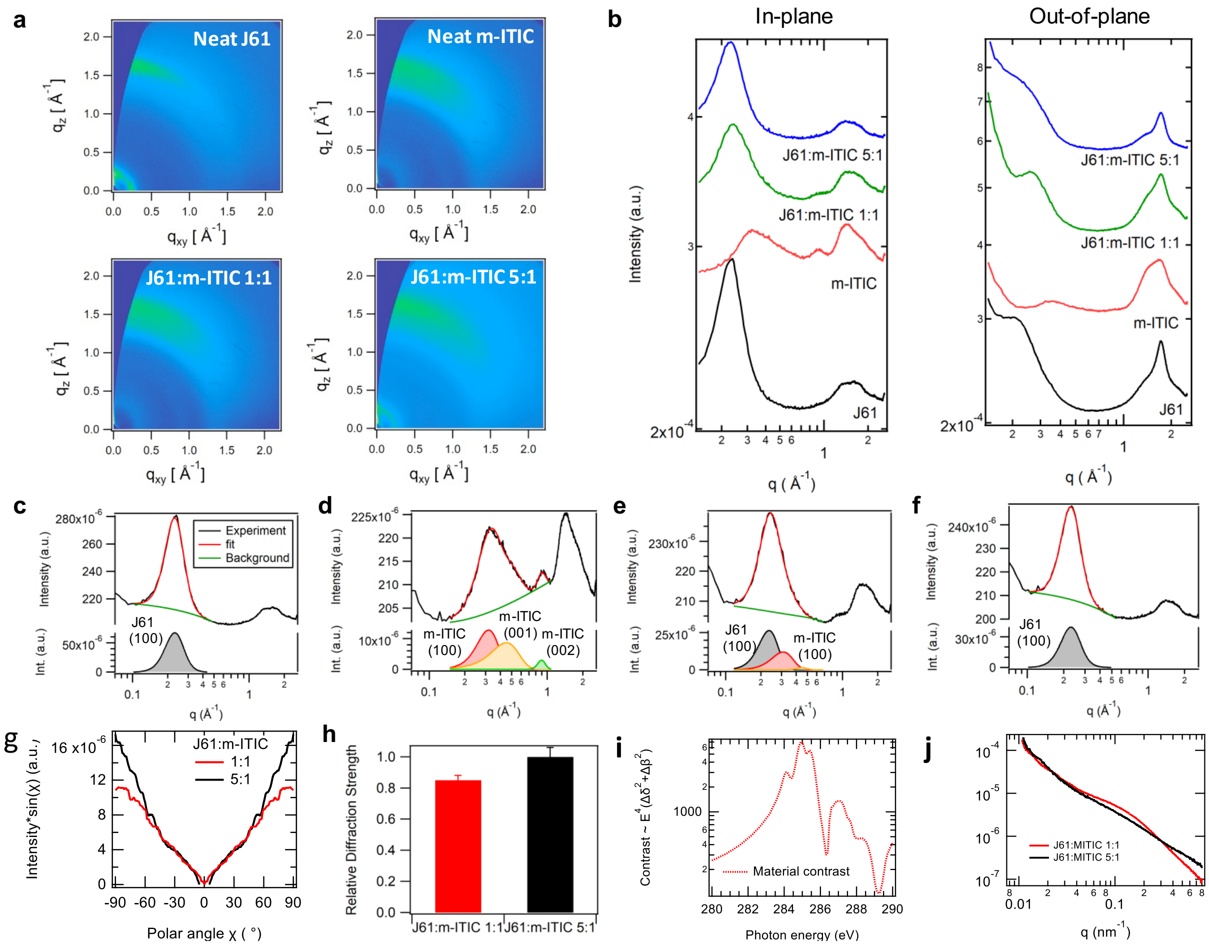

Supplementary Figure 5. **GIWAXS and R-SoXs measurements. a.** 2D Grazing incident wide angle X-ray scattering (GIWAXS) data for neat J61**,** neat m-ITIC, J61:m-ITIC (1:1) BHJ and J61:m-ITIC (5:1) BHJ. **b.** 1D in-plane and out-of-plane profiles from 15° cake sectors. **c-f.** Peak fits to first order diffraction peaks in in-plane profiles for neat J61, neat m-ITIC, J61:m-ITIC (1:1) BHJ, and J61:m-ITIC (5:1) BHJ respectively. **g.** Scattering volume and J61 volume normalized pole figures obtained from J61 (100) peaks in GIWAXS data for 1:1 and 5:1 blends. **h.** Normalized relative diffraction strength obtained from integration of pole figures shown in **g**. **i.** R-SoXS material contrast between J61 and m-ITIC calculated from transmission NEXAFS data. **j.** Azimuthally averaged 1D R-SoXS profiles obtained from 2D R-SoXS data (not shown). Uncertaintly in relative diffraction strengths were estimated from uncertainties in thickness and X-ray footprint measurements on the samples.

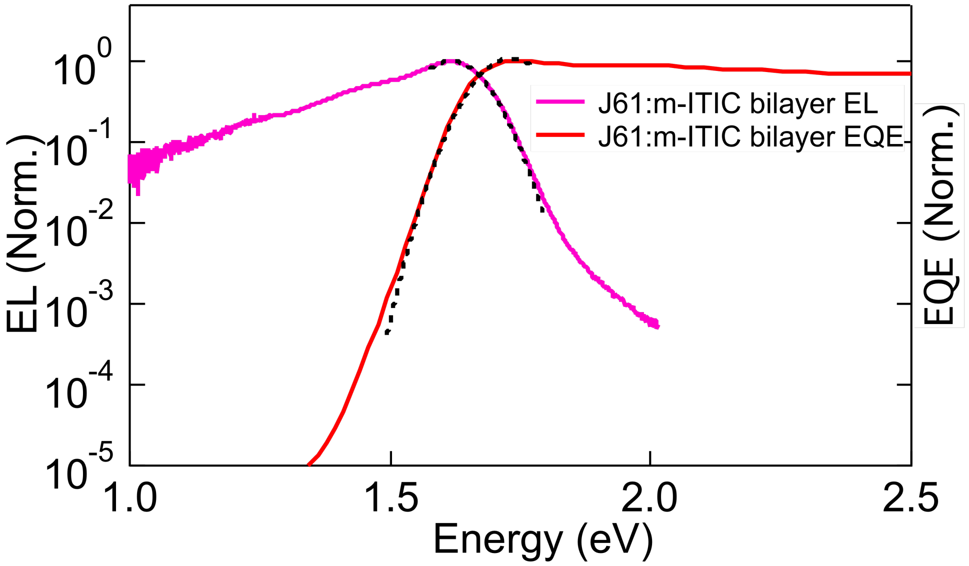

Supplementary Figure 6. **EL and EQE spectra of the J61:m-ITIC bilayer.** Due to limited interfacial area in the planar heterojunction, the CT response is too low to be detected, leaving only the S_1 state visible in the spectra.

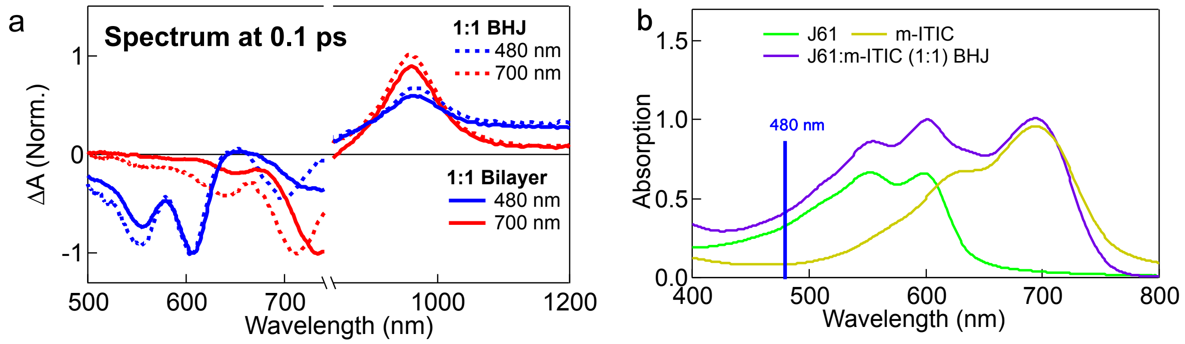

Supplementary Figure 7. **Early TA and steady-state absorption spectra of the J61:m-ITIC BHJ and bilayer. a**. TA spectra of the J61:m-ITIC (1:1) BHJ and bilayer at 0.1 ps under 480 and 700 nm excitation. **b**. Absorption spectrum of the J61:m-ITIC (1:1) BHJ and constituting neat J61 and neat m-ITIC spectra. The neat m-ITIC spectrum was manually blue-shifted to match the peak at 700 nm in the blend. Absorption by both m-ITIC and J61 is seen at 480 nm in the blend.

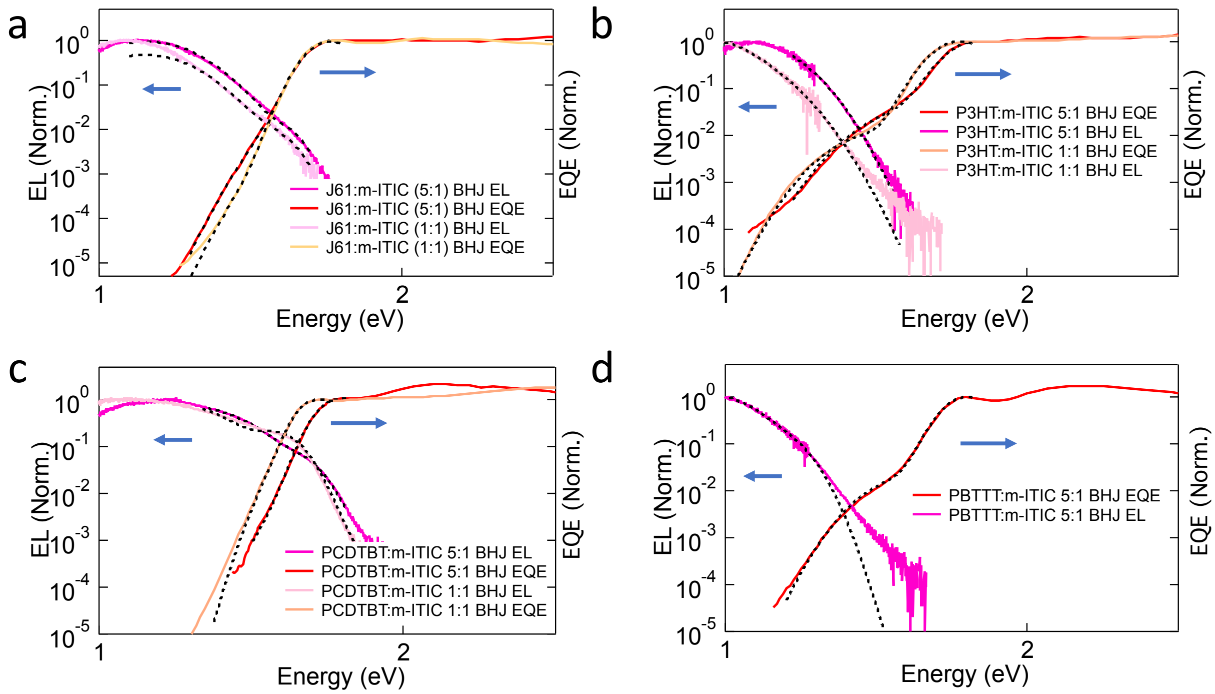

Supplementary Figure 8. **sEQE and EL measurements of polymer:m-ITIC blends.** The experimental spectra are shown together with the analysis of the sEQE and EL spectra with bi-Gaussian functions according to equations 1 and 2, allowing to extract the energy of the S_1 and CT states. Fitting parameters are listed in Supplementary Table 2.

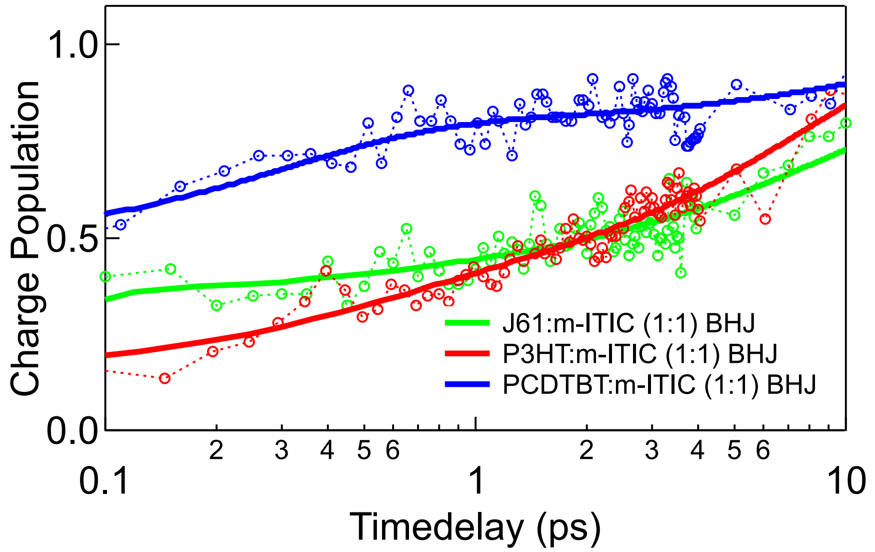

Supplementary Figure 9. **Charge rise upon 480 nm excitation in different samples.** Charge rise in J61:m-ITIC (1:1) BHJ, P3HT:m-ITIC (1:1) BHJ and PCDTBT:m-ITIC (1:1) BHJ under 480 nm excitation. A population of charges formed within the time resolution of the experiment is seen for all samples.

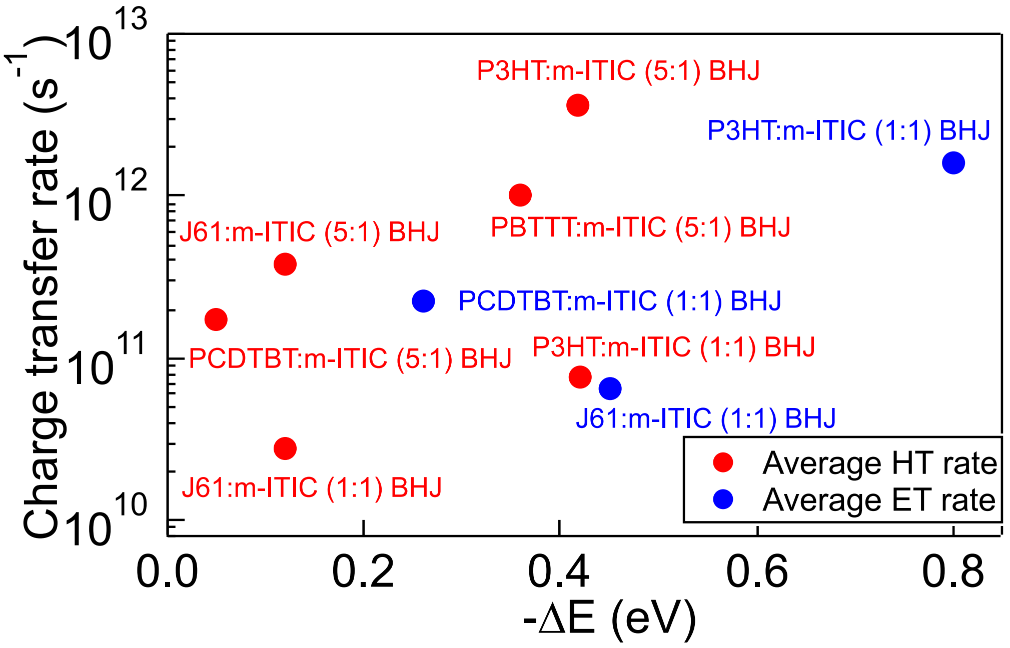

Supplementary Figure 10. **Average charge-transfer rates versus driving forces.** Inverse of average charge rise time with 700 nm excitation (red) and 480 nm excitation (blue) against driving force of hole transfer (700 nm) or electron transfer (480 nm). See Table 1 in the main text for the data. The random scatter of the data at 700 nm (exclusively HT) is due to the arbitrary contribution of exciton diffusion through the complex morphology of the blends, especially for the 1:1 samples. In the 5:1 blends, the average HT rate is dominated by the short time constant, where exciton diffusion is absent. The slower time constant contributing to the average could be due to exciton diffusion through residual aggregates, or due to donor:acceptor pairs with a less favorable molecular geometry and coupling. For 480 nm excitation, the charge rise is determined by an intricate mixture of electron transfer, hole transfer and excitation energy transfer.

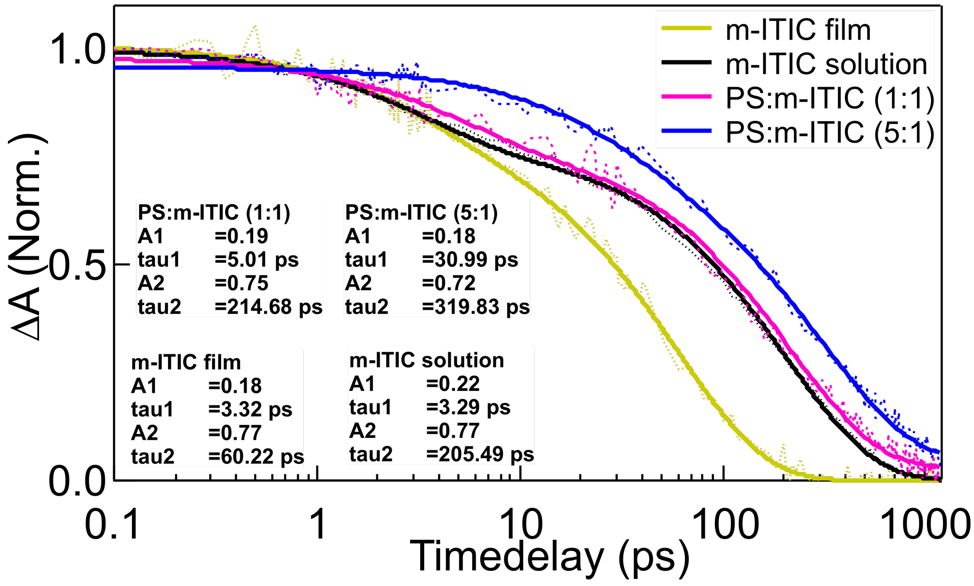

Supplementary Figure 11. **TA dynamics of the S_1 excited state absorption in m-ITIC.** Lifetime of the S_1 state in neat m-ITIC film, m-ITIC solution, PS:m-ITIC (1:1) and PS:m-ITIC (5:1), excited at 700 nm and probed in the ESA peak at 960 nm. The decay is generally multiphasic, depending on the packing and environment of the m-ITIC molecules. A first decay time constant of 3.3 ps is obtained in the neat film and solution, meaning that some competition with slower diffusion-mediated HT might occur in polymer blends containing m-ITIC domains.

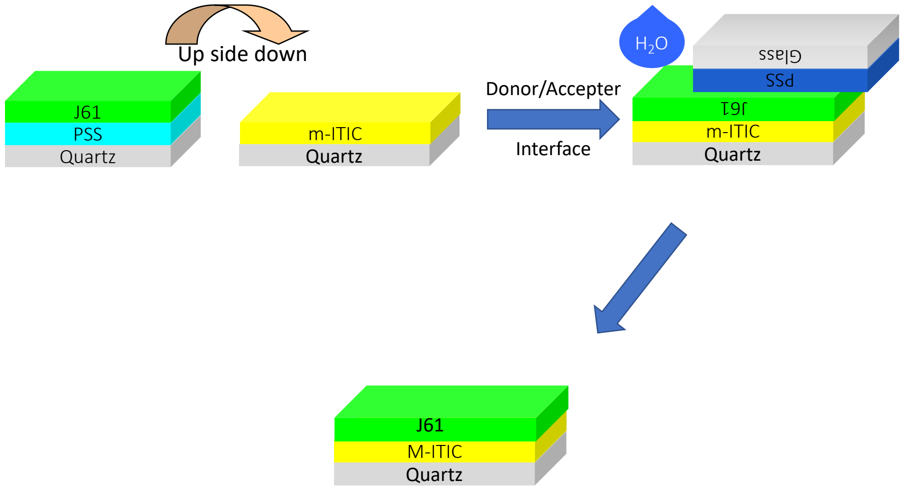

Supplementary Figure 12. **Lamination method for making the bilayer samples.** The glass:PSS:J61 sample was placed upside down onto a m-ITIC-coated quartz substrate. A drop of water placed on the edge of these two substrates dissolved the PSS, resulting in the transfer of the J61 layer onto the m-ITIC layer.

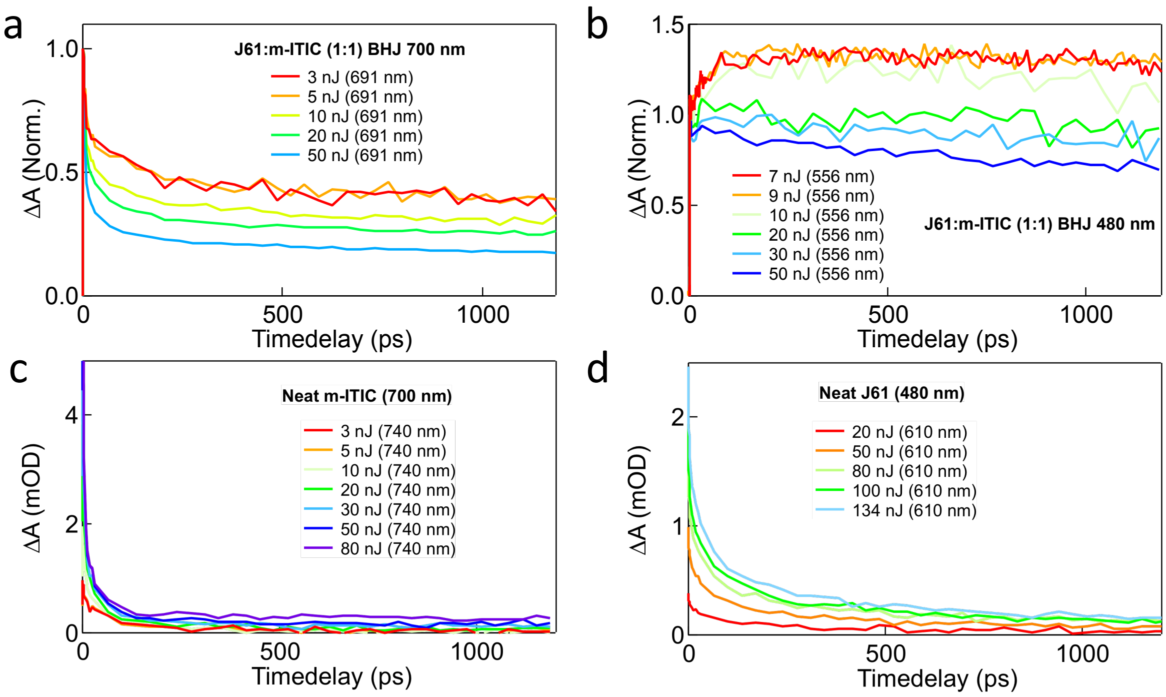

Supplementary Figure 13. Fluence-dependent TA measurements. a. b. Normalized fluence-dependent dynamics (probe wavelength labeled in the figure) for the J61:m-MITIC samples, showing the absence of higher-order effects at the used fluence of 3 and 7 nJ for 700 nm and 480 nm excitation, respectively. **c. d.** Fluence-dependent dynamics of neat m-ITIC (3 nJ used) and J61 (20 nJ used) films. The initial intensity was used to calculate the extinction coefficient (see Supplementary Figure 10).

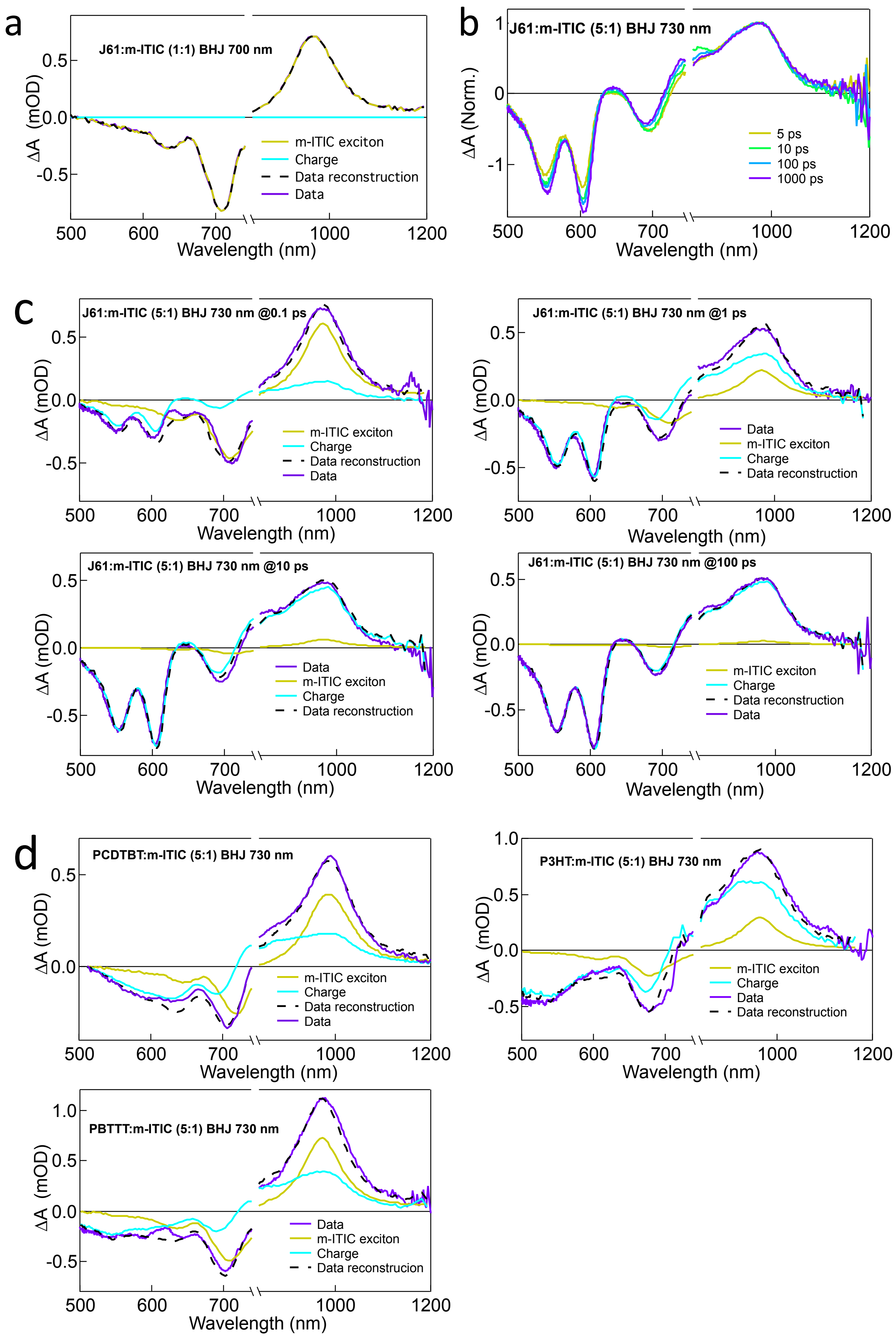
a
J61:m-ITIC (1:1) BHJ 700 nm
m-ITIC exciton
Charge
Data reconstruction
Data
ΔA (mOD)
Wavelength (nm)
b
J61:m-ITIC (5:1) BHJ 730 nm
5 ps
10 ps
100 ps
1000 ps
ΔA (Norm.)
Wavelength (nm)
c
J61:m-ITIC (5:1) BHJ 730 nm @0.1 ps
J61:m-ITIC (5:1) BHJ 730 nm @1 ps
J61:m-ITIC (5:1) BHJ 730 nm @10 ps
J61:m-ITIC (5:1) BHJ 730 nm @100 ps
d
PCDTBT:m-ITIC (5:1) BHJ 730 nm
P3HT:m-ITIC (5:1) BHJ 730 nm
PBTTT:m-ITIC (5:1) BHJ 730 nm
Data reconstrucion

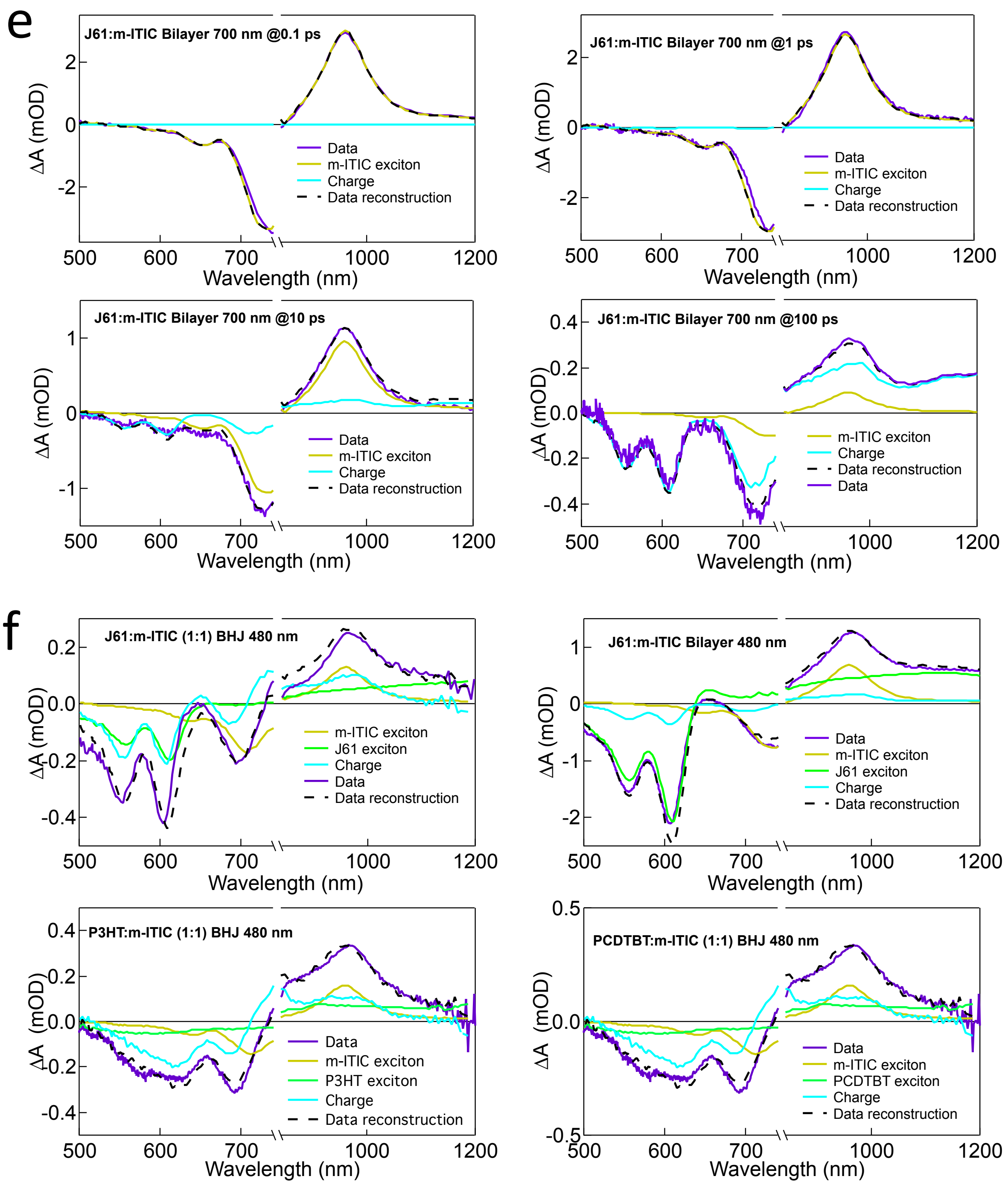

Supplementary Figure 14. **Reconstruction of TA spectra with sum of spectral components. a.** Reconstruction of the TA spectrum at 0.1 ps for the J61:m-ITIC (1:1) BHJ under 700 nm excitation, which contains only the contribution of the m-ITIC exciton. **b**. To verify that there is no intermediate state present (such as a hybridized S_1/CT state), we plot the normalized TA spectra of the J61:m-ITIC (5:1) BHJ under 730 nm excitation at 5, 10, 100 and 1000 ps (time delays at which the charge transfer is almost complete), and indeed no significant spectral changes, except a weak contribution of delayed hole transfer, are seen. **c.** Additionally, we show the data reconstruction of the J61:m-ITIC (5:1) BHJ under 730 nm excitation at 0.1, 1, 10, 100 ps, where the reconstructed data matches the experimental data well, evidencing validity of our spectral decomposition (using only the two components of the m-ITIC exciton and charge signatures). **d.**

Reconstruction of the TA spectrum at 0.1 ps for the different donor:m-ITIC 5:1 blends under 730 nm excitation, always using two components. **e.** Data reconstruction of the J61:m-ITIC bilayer under 700 nm excitation at 0.1, 1, 10 and 100 ps, also showing a successful decomposition into the m-ITIC exciton and charge components. **f.** Decomposition of the 0.1 ps TA spectrum of different 1:1 BHJ and bilayer samples under 480 nm excitation into a linear combination of m-ITIC and polymer excitons and charges (3 components).

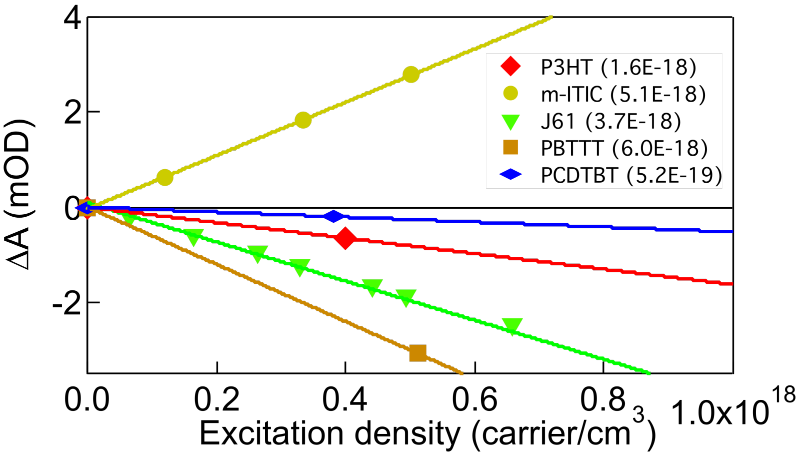

Supplementary Figure 15. **Determination of the extinction coefficient of the neat films from the initial TA signal intensity.** Initial TA intensity of the GSB signals in the neat polymers (e.g. at 556 nm for J61) and of the ESA peak (960 nm) in neat m-ITIC film as a function of excitation density. The extinction coefficient for the excitons is deduced from the slope and given in the legend (in cm^3).

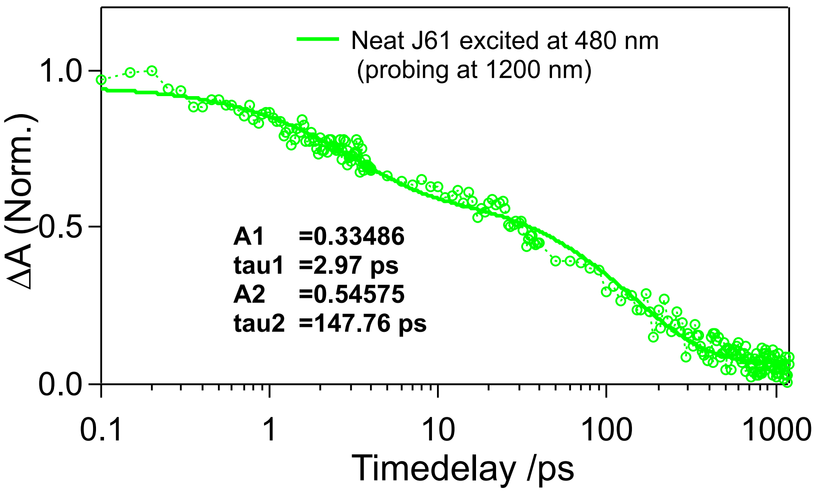

Supplementary Figure 16. **TA dynamics of neat J61 film**. S_1 lifetime of J61 (neat film) probing at 1200 nm under 480 nm excitation.

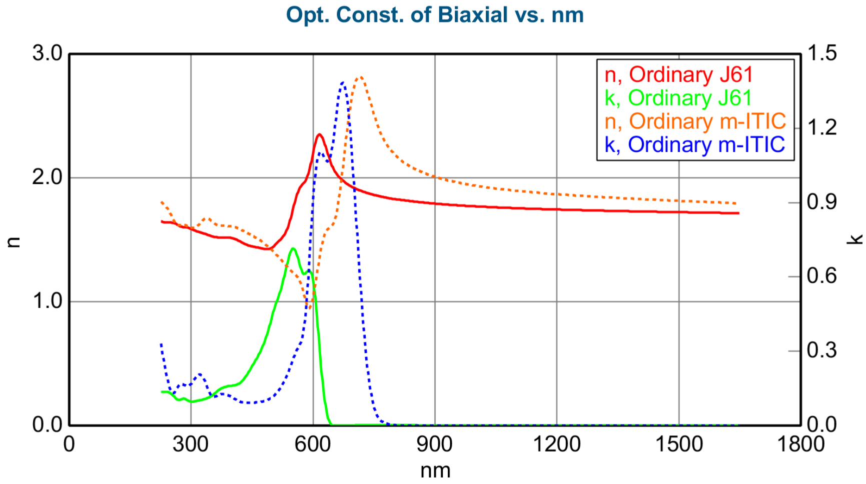

Supplementary Figure 17. **Spectroscopic ellipsometry of neat m-ITIC and J61 films**. In-plane complex index of refraction obtained from spectroscopic ellipsometry measurements of a ≈200 nm thick J61 film (solid lines) and 130 nm thick m-ITIC film (dotted lines). A uniaxial layer, with a Kramers-Kronig consistent b-spline functional form for both ordinary (in-plane) and extraordinary (out-of-plane, not shown) was assumed.

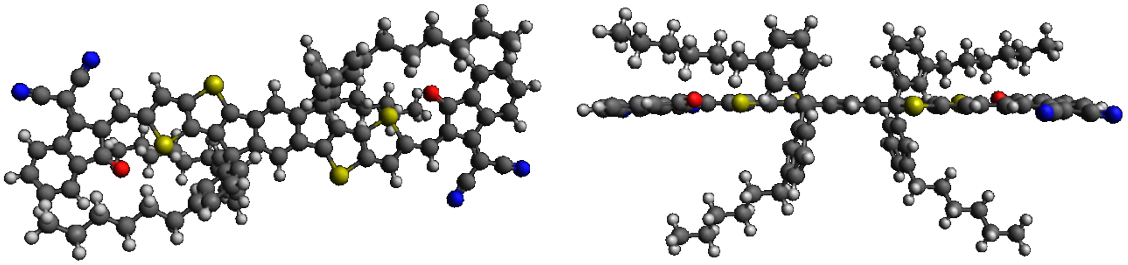

Supplementary Figure 18. **DFT structural modelling of m-ITIC.** Top and side view of m-ITIC model structure optimized using the B3LYP/6-21G level of theory (see Methods section in the main text).

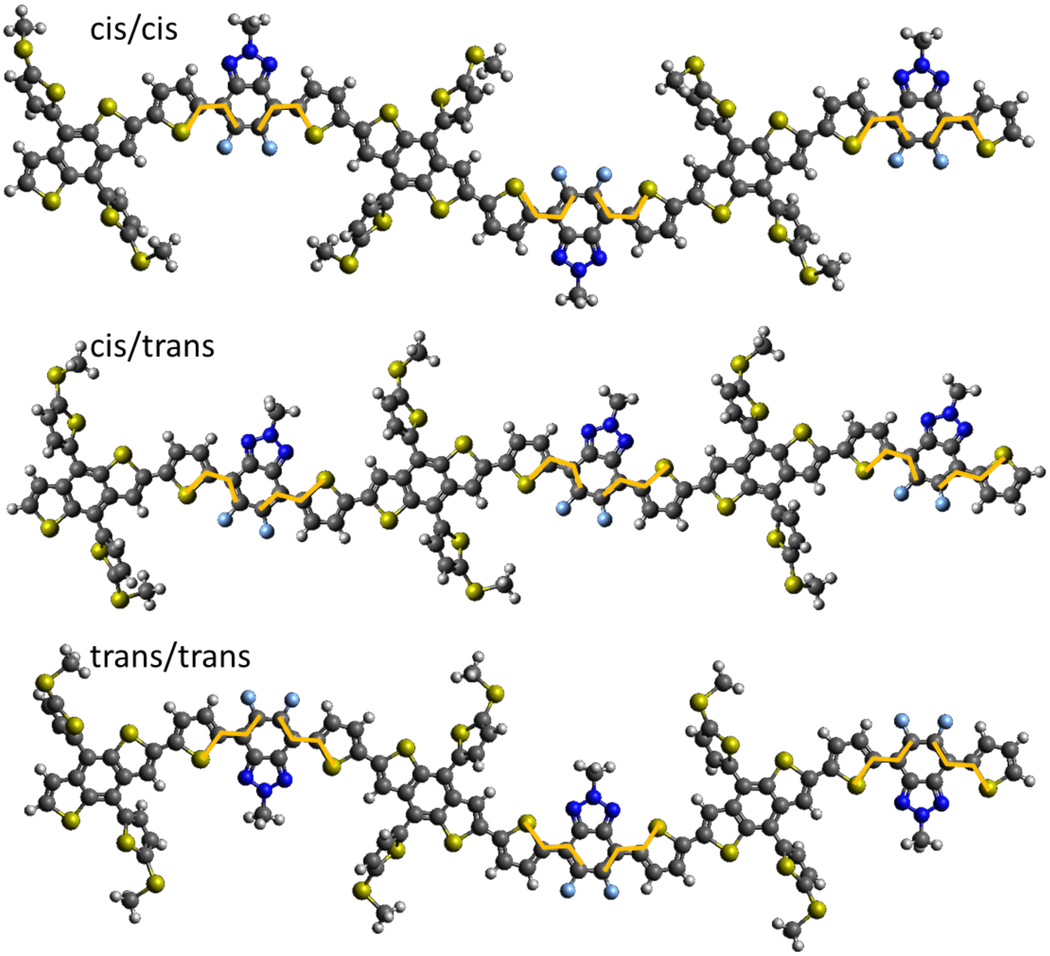

Supplementary Figure 19. **DFT structural modelling of J61.** Top view to the J61 structure models in cis/cis, cis/trans and trans/trans geometry. The bonds indicating the orientation of adjacent thiophene and benzotriazole units, are highlighted.

Supplementary Table 1. Time constants obtained by multi-exponential analysis (convoluted with a Gaussian instrument response function) of the exciton decay components (positive amplitudes) and charge rise component (negative amplitudes) obtained from the spectral decomposition of the TA spectra.

**Sample**	**Excitation (nm)**	**Component**	τ_1 **(ps)**	τ_2 **(ps)**	τ_3 **(ps)**	τ_4 **(ps)**
J61:m-ITIC (1:1)	480	m-ITIC exciton	64 (100 %)			
		J61 exciton	0.46 (37 %)	7.4 (63 %)		
		Charge	<0.06 (-34 %)	0.51 (-5 %)	8.4 (-39 %)	57 (-22 %)
P3HT:m-ITIC (1:1)	480	m-ITIC exciton	5.9 (56 %)	46 (44 %)		
		P3HT exciton	0.46 (44 %)	21 (56%)		
		Charge	<0.06 (-13 %)	0.47 (-18 %)	8.8 (-69 %)	
PCDTBT:m-ITIC (1:1)	480	m-ITIC exciton	1.6 (58 %)	341 (42 %)		
		PCDTBT exciton	0.06 (77 %)	17.4 (23 %)		
		Charge	<0.06 (-40 %)	0.3 (-35 %)	17.4 (-25 %)	
J61:m-ITIC (1:1)	700	m-ITIC exciton	0.78 (26 %)	12.2 (40 %)	82 (34 %)	
		Charge	0.78 (-14 %)	12.2 (-51 %)	82 (-35 %)	
J61:m-ITIC bilayer	700	m-ITIC exciton	0.92 (13 %)	9.1 (36 %)	63 (51 %)	
		Charge	0.92 (-17 %)	46 (-83 %)		
J61:m-ITIC (5:1)	730	m-ITIC exciton	0.40 (79 %)	7.8 (14 %)	200 (8 %)	
		Charge	0.40 (-70 %)	7.8 (-30 %)		
PBTTT:m-ITIC (5:1)	730	m-ITIC exciton	0.16 (73 %)	4.0 (27 %)		
		Charge	0.16 (-78 %)	4.0 (-22 %)		
P3HT:m-ITIC (5:1)	730	m-ITIC exciton	0.08 (99.7 %)	2.6 (0.03 %)		
		Charge	0.08 (-92 %)	2.6 (-8 %)		
PCDTBT:m-ITIC (5:1)	730	m-ITIC exciton	0.40 (87 %)	13.3 (13 %)		
		Charge	0.40 (-57 %)	13.3 (-43 %)		

Supplementary Table 2. Bi-Gaussian fitting parameters of the sEQE/EL spectra: Optical bandgap of m-ITIC (E_{opt}), corresponding reorganization energy (λ_{opt}); CT state energy (E_{CT}) and corresponding reorganization energy (λ_{CT})

**Sample**	E_{opt} **(eV)**	λ_{opt} **(eV)**	E_{CT} **(eV)**	λ_{CT} **(eV)**
**PBTTT:m-ITIC (5:1) BHJ**	1.70	0.10	1.34	0.26
**P3HT:m-ITIC (5:1) BHJ**	1.69	0.14	1.27	0.36
**PCDTBT:m-ITIC (5:1) BHJ**	1.68	0.06	1.63	0.48
**J61:m-ITIC (5:1) BHJ**	1.68	0.06	1.56	0.40
**P3HT:m-ITIC (1:1) BHJ**	1.64	0.14	1.20	0.29
**J61:m-ITIC (1:1) BHJ**	1.67	0.09	1.55	0.40
**PCDTBT:m-ITIC (1:1) BHJ**	1.68	0.07	1.62	0.25
**J61:m-ITIC Bilayer**	1.65	0.07	/	/
**Neat m-ITIC**	1.65	0.07	/	/

Supplementary Table 3. Initial (0.1 ps) charge yield and exciton populations of the BHJs under 480 nm excitation.

	**Polymer exciton**	**m-ITIC exciton**	**charge**
**P3HT:m-ITIC (1:1)**	46 %	34 %	20 %
**P3HT:m-ITIC (bilayer)**	59 %	25 %	16 %
**J61:m-ITIC (1:1)**	36 %	28 %	36 %
**PCDTBT:m-ITIC (1:1)**	37 %	13 %	50 %

Supplementary Table 4. Morphological information extracted from GIWAXS. Lamellar (100) spacings and coherence lengths obtained from peak fits to GIWAXS profiles as shown in Supplementary Figure 5. Uncertainties are standard deviations from the peak fits.

Sample	Peak	Spacing (nm)	Coherence length (nm)
**Neat J61**	J61 (100)	2.7	5.9 ± 0.1
**Neat m-ITIC**	m-ITIC (100)	2.0	3.6 ± 0.1
**J61:m-ITIC 1:1**	J61 (100)	2.7	5.4 ± 0.1
	m-ITIC (100)	2.0	3.7 ± 0.2
**J61:m-ITIC 5:1**	J61 (100)	2.7	5.9 ± 0.1

Supplementary Table 5. n and k values obtained for m-ITIC and J61 films from ellipsometry. Uncertainties are standard deviation from model fits to experimental data. The index of refraction of glass is taken as 1.506 and 1.516 at 700 and 480 nm respectively.

**Sample**	**Wavelength (nm)**	***n***	***k***	**Fraction absorbed light**
m-ITIC	480	1.471 ± 0.005	0.098 ± 0.004	0.019
	700	2.728 ± 0.027	0.999 ± 0.012	0.271
J61	480	1.426 ± 0.002	0.336 ± 0.001	0.064
	700	1.920 ± 0.001	0 ± 0.001	0.000

Supplementary Table 6. Parameters from DFT calculation. Total energy differences of the molecular structure models for J61 and m-ITIC in neutral ($\boldsymbol{R_0}$) and charged ($\boldsymbol{R_\pm}$) equilibrium geometry relative to the most stable configuration found and reorganization energy for charge transfer ($\boldsymbol{\lambda_{0\to\pm}}$). Values were obtained from B3LYP/6-21G calculations and are given in meV.

**J61**				**m-ITIC**	
	cis/cis	cis/trans	trans/trans		
$\boldsymbol{\Delta E(R_0)}$	-	126	245	$\boldsymbol{\Delta} E(\boldsymbol{R_0})$	-
$\boldsymbol{\Delta E(R_+)}$	56	183	300	$\boldsymbol{\Delta} E(\boldsymbol{R_-})$	105
$\boldsymbol{\lambda_{0\to+}}$	56	58	54	$\lambda_{0\to-}$	105

Supplementary Note 1

Analysis of the TA data (spectral decomposition)

We observe three main components in the TA spectra of the investigated donor:acceptor bilayers and blends, namely the spectral signatures of the acceptor excitons (m-ITIC S_1 state), of the donor excitons and of the photogenerated charges. The former two were identified by comparison to the neat m-ITIC and polymer films (Supplementary Figure 3), while the latter was seen in the TA spectra at long time delays when charge transfer was complete. In order to separately follow the time evolution of the different exciton and charge contributions, the TA spectra at all time delays were decomposed into a linear combination of their components using a linear least square fitting procedure.[1] Since knowing the spectral components is a pre-requisite of this analysis, the singlet exciton spectrum of the neat polymers was taken from their TA spectrum at 0.1 ps, the singlet exciton spectrum of m-ITIC was extracted from the TA spectrum of the neat acceptor film or of the J61:m-ITIC (1:1) BHJ at 0.1 ps, and the charge signature for all the heterojunctions was extracted from the TA data at a long time delay (usually 1ns). The early J61:m-ITIC (1:1) BHJ signal at 700 nm excitation was used as signature of the m-ITIC exciton to analyze the other BHJ blends, to account for the blue-shift of the m-ITIC ground state bleaching (GSB) and simulated emission (SE) compared to the neat m-ITIC film, in view of the shift already observed in the steady-state absorption spectra. This is reasonable, since we showed that there is no direct J61 excitation nor prompt charge transfer in the 1:1 BHJ at 700 nm. An additional shift of a few nanometers was introduced if necessary.

Supplementary Figure 14 shows examples of the spectral decomposition for the TA spectra of the different samples excited both at 480 nm and 700 nm, including the scaled components and the spectrum reconstructed as their linear combination. The time evolution of the components was obtained by plotting the coefficients of the fitting procedure as a function of time delay. To exclude the presence of an intermediate state (e.g. due to S_1/CT hybridization) and the validity of using only two spectral components (m-ITIC exciton and charges) under 700/730 nm excitation, we have normalized the TA spectra of the 5:1 J61:m-ITIC blend at 5, 10, 100 and 1000 ps (when almost only charges are present) (Supplementary Figure 14b). No spectral evolution is observed, indicating that a single charge component can be used to analyze the spectra (no intermediate state is detected). Moreover, we show that the spectral reconstruction of the J61:m-ITIC 5:1 and bilayer samples with 700 nm excitation at 0.1, 1, 10 and 100 ps can be achieved with only one exciton and one charge component (Supplementary Figure 14c,e). Since the reconstructed data matches the experimental data well, this suggests validity of our spectral decomposition (without additional components).

To relate the amplitude of the decomposition coefficients to the population of the excitons and charges, the extinction coefficients of the species were determined at a given probe wavelength. To do this, we plotted the TA signal intensity of the neat m-ITIC film probed at 960 nm (excited states absorption (ESA) band) at 0.1 ps, and the intensity of the GSB signal of the neat polymer films (e.g. 556 nm for J61) at 0.1 ps as a function of excitation density (Supplementary Figure 15). The concentration of excitons at 0.1 ps is equal to the density of absorbed photons (no recombination to the ground state has taken place yet) and so that we used the slope of the graphs as a measure of the extinction coefficient. From this, we calculated the population of the different excitons in the 0.1 ps TA spectra, using the intensity of the exciton components from the decomposition. For the charges, we assumed 100 % conversion of absorbed photons to charges (counting one electron/hole pair per photon) at a time delay where charge transfer is complete and

before recombination sets in for all the BHJ samples, and then related the signal strength around 960 nm back to the percentage of charge population at 0.1 ps (if pertinent). In the J61:m-ITIC bilayer with 700 nm excitation, we estimated a total charge yield of 55% by comparing the initial m-ITIC exciton peak to the maximum charge peak at 960 nm. We could not determine a unique extinction coefficient for charges in all samples, since the signal intensity of the hole polarons is expected to change for the different polymers. The population of all excitons and charges at 0.1 ps found by this procedure was always of the order of 10^{17} cm^{-3} and matched the density of absorbed photons (from the excitation fluence and film thickness) quite well. We expressed the yield of the three species at 0.1 ps as a percentage of the total initial carrier concentration (see Table S3 for samples with 480 nm excitation) and scaled the dynamics traces of the spectral components at 0.1 ps to these values in order to express the populations of a percentage of the total absorbed photon density. Finally, those dynamics were analyzed using the convolution of a Gaussian-shaped instrument response function (IRF) with the sum of exponential terms (Supplementary Table 1). The analytical expression for this function has been described elsewhere.[2] The width of the IRF was found to be around 60 fs. Whenever applicable, the rise of the charges was globally fitted together with the decay of the excitons, i.e. by linking the corresponding time constants.

Supplementary Note 2

X-ray scattering – GIWAXS and R-SoXS

To verify that the m-ITIC in the 5:1 blend is molecularly dispersed in a J61 matrix, we compared morphology measurement results from grazing incidence wide angle X-ray scattering (GIWAXS) and resonance soft X-ray scattering (R-SoXS) of J61:m-ITIC 1:1 and 5:1 blends as shown in Supplementary Figure 5 and Supplementary Table 4. Deconvolution of strong (100) in-plane peaks reveal the presence of lamellar stacking of the polymer (≈ 0.23 $Å^{-1}$) as well as the m-ITIC (≈ 0.32 $Å^{-1}$) in the 1:1 sample, whereas only the polymer lamellar stacking peaks were observed for the 5:1 sample. This result indicates that the m-ITIC is not crystallized in the 5:1 sample, verifying that it is amorphous and/or molecularly dispersed in the polymer. Polymer (J61) coherence lengths were found to be similar between the blends and neat films (Supplementary Table 4), indicating similarly-sized regions of regular polymer order. Pole figures for the J61 (100) peak show that orientation distribution of ordered fractions and diffraction strengths of the polymer in the two samples are also very similar. Complementary information from R-SoXS further proves that the m-ITIC is molecularly dispersed in polymer. The R-SoXS scattering profile at a high material contrast energy (284 eV) shows a peak that corresponds to a characteristic phase separation size scale of ≈ 50 nm for the 1:1 blend. In contrast, the 5:1 blend exhibits an absence of any scattering feature, which allows us to rule out morphologies where amorphous m-ITIC is separated into domains with size scales larger than our measurement resolution (≈ 4 nm). Taken together, the GIWAXS and R-SoXS results show unambiguously that the m-ITIC is not crystalline and it is finely dispersed in a polymer matrix.

Supplementary Note 3

Analysis of the EQE/EL spectra

The reduced EQE/EL spectra were analyzed with a bi-Gaussian function to extract the values of the optical bandgap (E_{opt} = excited state S_1 energy of the acceptor compared to the ground state) and charge transfer (CT) state energy (E_{CT}). Fits of Eq. 1 and Eq. 2 were performed to the sEQE and EL data. (Supplementary Figure 8). E_{CT} and E_{opt} were obtained by fitting both EQE and EL data with bi-Gaussian dependencies following Eq.1 and Eq.2.

$$A(E) = \frac{f_A E}{\sqrt{4\lambda_{opt} k_B T}} \exp\left(-\frac{\left(E - E_{opt} - \lambda_{opt}\right)^2}{4\lambda_{opt} k_B T}\right) + \frac{f_A E}{\sqrt{4\lambda_{CT} k_B T}} \exp\left(-\frac{(E - E_{CT} - \lambda_{CT})^2}{4\lambda_{CT} k_B T}\right) \quad \textit{(eq. 1)}$$

$$N(E) = \frac{f_n E}{\sqrt{4\lambda_{opt} k_B T}} \exp\left(-\frac{\left(E - E_{opt} + \lambda_{opt}\right)^2}{4\lambda_{opt} k_B T}\right) + \frac{f_n E}{\sqrt{4\lambda_{CT} k_B T}} \exp\left(-\frac{(E - E_{CT} + \lambda_{CT})^2}{4\lambda_{CT} k_B T}\right) \quad \textit{(eq. 2)}$$

Here, E corresponds to the photon energy and k_B is the Boltzmann constant; E_{CT} is the energy for CT states, and E_{opt} is the energy for S_1; f_A and f_N are the pre-factors and they do not dependent on E and are proportional to the square of the electronic coupling matrix element.[3] The line-width is proportional to the temperature T and the low frequency relaxation energy λ_{opt} (λ_{CT}).

To obtain the driving force for ET, we calculated the bandgap of the polymers as the crossing of the normalized absorption and emission spectra (Supplementary Figure 2 and Supplementary Figure 3). Shifts of the polymer absorption in the blends compared to the neat film were generally small, but if they occurred, the bandgap was corrected accordingly. For example in PCDTBT, the absorption in the blend red shifts by 18 nm, so that the bandgap accordingly equals the bandgap of the neat polymer plus 0.014 eV. We found polymer S_1 energies of 2.00 eV for J61 and P3HT, of 1.88 eV for PCDTBT.

Supplementary Note 4

Kinetic modeling of the TA dynamics of J61:m-ITIC (1:1) BHJ under 480 and 700 nm excitation

700 nm excitation

At this excitation wavelength, only m-ITIC (the acceptor, A) is excited and the dynamics show a concomitant multi-phasic decay of m-ITIC excitons and rise of charges (C), caused by complex exciton diffusion over various distances within the phase morphology of the blend, followed by interfacial exciton dissociation. We note that since the TA signatures of charges in the CT state and of free charges are similar, we only used a single charge component to account for both of them (see also spectral decomposition in section 1.6). The dissociation of the CT state is therefore not treated in the kinetic model. Any energy transfer from an excited acceptor to the donor can be neglected due to unfavorable energetics. To model the exciton dissociation with a reasonable number of parameters, we used a single exponential with an average lifetime of 20 ps for the hole transfer process. An exciton lifetime of 160 ps (average of the m-ITIC solution measurement) was used, and a 20 ns charge recombination component (average of the 700 ps geminate recombination and slower bimolecular components outside of the time window) was introduced (electron back-transfer to the S_1 state was not accounted for). The shape of the dynamics is reasonably well reproduced (main text Figure 2, which also summarizes the main processes in a Jablonki diagram). Discrepancies in the fastest and slowest hole transfer components are due to neglecting the multiphasic diffusion-limited nature of the process. We note that with the used time constants, the model predicts a 12% loss of excitons to the ground state.

Parameters:
$k_{HT} = k1 = 1/20\ ps^{-1}$ (average hole transfer rate)
$k_{S1,A} = k2 = 1/160\ ps^{-1}$ (natural m-ITIC S_1 lifetime)
$k_{rec} = k3 = 1/20000\ ps^{-1}$ (average recombination rate)
a0 = 1 (initial acceptor population)
c0 = 0 (initial charge population)

Rate equations and solutions:
$\frac{d[A]}{dt} = -k1[A] - k2[A]$ *(eq. 3)*
→ $[A] = a0 * \exp(-t * (k1 + k2))$ *(eq. 4)*
$\frac{d[C]}{dt} = k1[A] - k3[C] = k1 * (a0 * \exp(-t * (k1 + k2))) - k3[C]$ *(eq. 5)*
→ $[C] = (a0 * k1 * \exp(-k3 * t))/(k1 + k2 - k3) - (a0 * k1 * \exp(-k1 * t) * \exp(-k2 * t))/(k1 + k2 - k3)$ *(eq. 6)*

480 nm excitation

At this excitation wavelength, both m-ITIC (A) and J61 (C) are excited and the ensuing processes are summarized in Figure 2 of the main text. Excited J61 decays by electron transfer and energy transfer to m-ITIC and by natural decay to the ground state. For the energy transfer (EET), we assume an average time constant of 6 ps, which corresponds to a donor-acceptor distance of about 3.3 nm (with a Förster radius of 5.2 nm). For the natural lifetime, we use 100 ps (average of neat J61 film data) and for the electron transfer 20 ps (this corresponds only to the slower diffusion-mediated process, since prompt electron transfer occurs faster than the time resolution of the

experiment). These time constants allow to well reproduce the overall decay of J61 excitons (main text Figure 2). Due to the prompt electron transfer, the initial charge population is 36% (deduced from decomposition of the 100 fs TA spectrum). We assume that those charges are generated only by electron and not by hole transfer, since no ultrafast hole transfer is observed with 700 nm excitation. Initially, there are also 36% J61 excitons and 28% m-ITIC excitons present, the latter corresponding well to the absorbance of m-ITIC at 480 nm. This implies that m-ITIC is directly excited and not populated by energy transfer within the time resolution of the experiment (i.e. at ultrafast times, electron transfer from J61 outcompetes energy transfer). In the kinetic model, we assume that directly excited m-ITIC decays with an average 20 ps hole transfer rate and 160 ps natural decay rate, similar as with 700 nm excitation. On the other hand, m-ITIC excitons populated by slower energy transfer are already close to a donor interface and therefore undergo hole transfer only with the 0.8 ps intrinsic time constant (without exciton diffusion). Charges are generated by both electron and hole transfer and undergo weak recombination (20 ns time constant). The behavior of the dynamics is well reproduced with this model, except for minor discrepancies (especially on the HT rate) due to not accounting for multiphasic exciton diffusion. With the used time constants, the model predicts a 6% loss of excitons to the ground state.

Parameters:
k_{ET} =k1=1/20 ps^{-1} (average diffusion-limited electron transfer rate)
$k_{S1,D}$ = k2=1/100 ps^{-1} (natural J61 S_1 lifetime)
k_{EET} = k3=1/6 ps^{-1} (average energy transfer rate)
k_{HT} = k4=1/20 ps^{-1} (average hole transfer rate for directly excited m-ITIC)
$k_{S1,A}$ = k5 = 1/160 ps^{-1} (natural m-ITIC S_1 lifetime)
$k_{HT,EET}$ = k6=1/0.8 (hole transfer rate for excited m-ITIC populated by EET)
k_{rec} = k7 = 1/20000 ps^{-1} (average recombination rate)
a0 = 0.28 (initial acceptor population, directly excited)
d0 = 0.36 (initial donor population, directly excited)
c0 = 0.36 (initial charge population from prompt ET)

Rate equations and solutions:

$$\frac{d[D]}{dt} = -k1[D] - k2[D] - k3[D] \qquad \textit{(eq. 7)}$$

$$\rightarrow [D] = \mathrm{d0} * \exp\left(-\mathrm{t} * (\mathrm{k1} + \mathrm{k2} + \mathrm{k3})\right). \qquad \textit{(eq. 8)}$$

$$\frac{d[A]_{direct}}{dt} = -k4[A]_{direct} - k5[A]_{direct} \qquad \textit{(eq. 9)}$$ directly excited m-ITIC

$$\rightarrow [A]_{direct} = \mathrm{a0} * \exp\left(-\mathrm{t} * (\mathrm{k4} + \mathrm{k5})\right). \qquad \textit{(eq. 10)}$$

$$\frac{d[A]_{EET}}{dt} = -k6 * [A]_{EET} - k5 * [A]_{EET} + k3 * (d0 * exp(-t * (k1 + k2 + k3))); \qquad \textit{(eq. 11)}$$ EET

$$\rightarrow [A]_{EET} = (\mathrm{d0} * \mathrm{k3} * \exp(-\mathrm{t} * (\mathrm{k5} + \mathrm{k6})))/(\mathrm{k1} + \mathrm{k2} + \mathrm{k3} - \mathrm{k5} - \mathrm{k6}) - (\mathrm{d0} * \mathrm{k3} * \exp(-\mathrm{t} * (\mathrm{k5} + \mathrm{k6})) * \exp(-\mathrm{k1} * \mathrm{t}) * \exp(-\mathrm{k2} * \mathrm{t}) * \exp(-\mathrm{k3} * \mathrm{t}) * \exp(\mathrm{k5} * \mathrm{t}) * \exp(\mathrm{k6} * \mathrm{t}))/(\mathrm{k1} + \mathrm{k2} + \mathrm{k3} - \mathrm{k5} - \mathrm{k6}). \qquad \textit{(eq. 12)}$$

$$\rightarrow [A]_{total} = [A]_{direct} + [A]_{EET} = \mathrm{a0} * \exp\left(-\mathrm{t} * (\mathrm{k4} + \mathrm{k5})\right) + (\mathrm{d0} * \mathrm{k3} * \exp(-\mathrm{t} * (\mathrm{k5} + \mathrm{k6})))/(\mathrm{k1} + \mathrm{k2} + \mathrm{k3} - \mathrm{k5} - \mathrm{k6}) - (\mathrm{d0} * \mathrm{k3} * \exp(-\mathrm{t} * (\mathrm{k5} + \mathrm{k6})) * \exp(-\mathrm{k1} * \mathrm{t}) * \exp(-\mathrm{k2} * \mathrm{t}) * \exp(-\mathrm{k3} * \mathrm{t}) * \exp(\mathrm{k5} * \mathrm{t}) * \exp(\mathrm{k6} * \mathrm{t}))/(\mathrm{k1} + \mathrm{k2} + \mathrm{k3} - \mathrm{k5} - \mathrm{k6}. \qquad \textit{(eq. 13)}$$

$$\frac{d[C]}{dt} = k4[A]_{direct} + k6[A]_{EET} + k1[D] - k7[C] = k4 * (a0 * exp(-t * (k4 + k5))) + k6 * ((d0 * k3 * exp(-t * (k5 + k6)))/(k1 + k2 + k3 - k5 - k6) - (d0 * k3 * exp(-t * (k5 + k6)) *$$

$exp(-k1 * t) * exp(-k2 * t) * exp(-k3 * t) * exp(k5 * t) * exp(k6 * t))/(k1 + k2 + k3 - k5 - k6)) + k1 * (d0 * exp(-t * (k1 + k2 + k3))) - k7 * [C]$; *(eq. 14)*

→ $[C] = \mathrm{exp(-k7 * t) * (c0 - (d0 * k1\^{}2 + d0 * k1 * k2 + d0 * k1 * k3 - d0 * k1 * k5 - d0 * k1 * k6 - d0 * k3 * k6)/(k1 * k5 - 2 * k1 * k3 - 2 * k2 * k3 - 2 * k1 * k2 + k1 * k6 + k2 * k5 + k1 * k7 + k2 * k6 + k3 * k5 + k2 * k7 + k3 * k6 + k3 * k7 - k5 * k7 - k6 * k7 - k1\^{}2 - k2\^{}2 - k3\^{}2) + (a0 * k4)/(k4 + k5 - k7) + (d0 * k3 * k6)/(k1 * k5 + k1 * k6 + k2 * k5 - k1 * k7 + k2 * k6 + k3 * k5 - k2 * k7 + k3 * k6 - k3 * k7 - 2 * k5 * k6 + k5 * k7 + k6 * k7 - k5\^{}2 - k6\^{}2)) - exp(-k7 * t) * ((a0 * k4 * exp(k7 * t - k5 * t - k4 * t))/(k4 + k5 - k7) - (exp(k7 * t - k2 * t - k3 * t - k1 * t) * (d0 * k1\^{}2 + d0 * k1 * k2 + d0 * k1 * k3 - d0 * k1 * k5 - d0 * k1 * k6 - d0 * k3 * k6))/(k1 * k5 - 2 * k1 * k3 - 2 * k2 * k3 - 2 * k1 * k2 + k1 * k6 + k2 * k5 + k1 * k7 + k2 * k6 + k3 * k5 + k2 * k7 + k3 * k6 + k3 * k7 - k5 * k7 - k6 * k7 - k1\^{}2 - k2\^{}2 - k3\^{}2) + (d0 * k3 * k6 * exp(k7 * t - k6 * t - k5 * t))/(k1 * k5 + k1 * k6 + k2 * k5 - k1 * k7 + k2 * k6 + k3 * k5 - k2 * k7 + k3 * k6 - k3 * k7 - 2 * k5 * k6 + k5 * k7 + k6 * k7 - k5\^{}2 - k6\^{}2))}$ *(eq. 15)*

Supplementary Note 5

Calculation for EET Förster Radius

We have calculated the Förster energy transfer (EET) transfer distance (Förster radius) to be around 5 nm, as shown below:

The rate of EET can be calculated from:

$$k_{EET} = \frac{1}{\tau_D}\left(\frac{R_0}{R}\right)^6 \qquad \textit{(eq. 16)}$$

where R is the distance between donor and acceptor molecules, τ_D is the fluorescence lifetime of the donor in the absence of the acceptor. The critical transfer distance (Förster radius) R_0 can be calculated using the relevant spectroscopic porperties of the participating molecules:

$$R_0 = 0.2108[\kappa^2 \Phi_0 n^{-4} J]^{\frac{1}{6}} \qquad \textit{(eq. 17)}$$

where κ^2 is the orientation factor, Φ_0 is the quantum yield of donor fluorescence (without acceptor), and n is the refractive index of the medium. J is the degree of spectral overlap between the donor fluorescence spectrum and acceptor absorption spectrum (scaled to its maximum molar extinction coefficient):

$$J = \int_0^\infty F_D\,(\lambda)\varepsilon_a(\lambda)\lambda^4 d\lambda \qquad \textit{(eq. 18)}$$

where F_D is the normalized donor fluorescence spectrum, ε_a is the extinction coefficient of the acceptor and λ is the wavelength.

ε_a can be calculated through the following:

$$A = \varepsilon_a c l \qquad \textit{(eq. 19)}$$

where A is the absorbance, c is the concentration and l is the thickness. We have measured the absorbance of m-ITIC (45 nm) by using an integrating sphere (see Supplementary Figure 2). Assuming a density of m-ITIC of 1 g cm^{-3}, we obtained ε_a of $1.01*10^7$ mol^{-1} cm^{-1} at 700 nm. Taking this value into formula (3), we obtained J of $1.7*10^{18}$ mol^{-1} cm^{-1} nm^4. By putting this value into formula (2), and assuming a dipole orientation factor of 0.66 (without knowing specific structural information), a refractive index of the medium of 1.5 (see *n* and *k* in TMM calculation), a quantum yield of J61 of 0.05, we obtain a critical distance R_0 of 5.2 nm. With this Förster radius, we use formula (1) to calculate a transfer rate of $1.7*10^{11}$ s^{-1} (time constant of 6 ps, used also in the kinetic modeling), by taking τ_D of average lifetime around 97 ps (see Supplementary Figure 16) and R of 3.3 nm (a reasonable average D:A distance in the BHJ).

Supplementary Note 6

Calculation of the excitation profiles in the bilayer

Transfer matrix calculations (TMM) were carried out to estimate the excitation profiles and exciton densities at the interface in the two bilayer experiments described in Figure 2 of the main text. The in-plane index of refraction (n+ik) for J61 and m-ITIC were obtained from variable angle spectroscopic ellipsometry (JA Woollam M-2000 XI) covering the wavelength range from 211 to 1650 nm and are shown in Supplementary Figure 17 and Supplementary Table 5. The transfer matrix calculation was performed with WVASE-32 software, with the parameters summarized in Supplementary Table 5. The calculation properly accounts for the reflection losses at the air:J61 and air:glass and glass:m-ITIC interfaces, and the internal multiple reflections. Light trapping effects are important due to the high refractive index of m-ITIC near the 700 nm resonance.

Supplementary Note 7

DFT structure modelling

For m-ITIC acceptor molecules (see Supplementary Figure 18), we consider the full molecular structure in the simulations, as in the course of our investigation it turned out that the position of the hexyl-phenyl sidechains of m-ITIC are important. Substituting the hexyl group by shorter chains was not possible because this led to the formation of hydrogen bonds between the alkyl hydrogens and the oxygen of the m-ITIC backbone. As a result, an artificial bending of m-ITIC was observed, which is absent in the full molecular structure, i.e. with hexyl groups. Furthermore, we found that when forming complex geometries with the J61 polymer, the hexyl-phenyl sidechains cause steric repulsion. For this reason, we consider the full m-ITIC molecule including the hexyl-phenyl sidechains in the simulations.

For the J61 polymer donor, we consider models with up to three repeat units that are terminated with hydrogen atoms. In order to save computational resources, the solubilizing sidechains have been partly substituted by methyl groups (see structures in Supplementary Figure 19). Since the alkyl chains mainly point away from the polymer backbone, their impact on the complex formation is minor. Moreover, they do not affect the frontier orbitals and can therefore be neglected. With this simplification, we simulate several polymer conformers. Since the individual units of the J61 backbone, in particular the thiophene rings and the benzotriazole unit, can be oriented in different ways, we consider cis and trans orientations between these units. Here, cis refers to the orientation where the thiophene Sulphur is on the same side as the Fluorine atoms, while in the trans orientation it is flipped to the other side (indicated in orange in Supplementary Figure 19).

To calculate the transfer integrals, donor-acceptor complexes are constructed by placing the m-ITIC on top of the J61 polymer. In doing so, it turned out, that positioning m-ITIC next to the benzotriazole unit suffers from steric repulsion between its hexyl side chains and the out-of-plane thiophenes of the polymer's donor unit. This leads to large distances (> 6 Å) for which the electronic coupling is negligibly small. Such geometries are therefore disregarded. In contrast, when m-ITIC is placed on top of the donor unit of J61, the molecules can come closer and only for distances of about 4.5 Å side groups start to bend away. The obtained complex is displayed in Figure 4b in the main text.

Supplementary Note 8

Calculation of MLJ plots

In order to describe the charge transfer, we use the semiclassical Marcus-Levich-Jortner (MLJ) model:[4, 5]

$$k_{\mathrm{MLJ}} = \frac{|J_{\mathrm{DA}}|^2}{\hbar}\sqrt{\frac{\pi}{\lambda_o k_{\mathrm{B}} T}}\sum_{l=0}^{3} F_l(S, N(\hbar\omega)) \exp\left[-\frac{(\lambda_o+\Delta E+l\hbar\omega)^2}{4\lambda_o k_{\mathrm{B}} T}\right] \qquad \textit{(eq. 20)}$$

with

$$F_l(S,N) = [(N+1)/N]^{l/2} \exp\,[-S(2N+1)] I_l\left(2S\sqrt{N(N+1)}\right) \qquad \textit{(eq. 21)}$$

and N and $I_l(x)$ being the Bose-Einstein distribution function and the modified Bessel functions, respectively. Herein, J_{DA} is the electronic coupling strength, $\lambda_o = 150$ meV is the outer reorganization energy. This parameter is assumed to be somewhat smaller than the intramolecular analog $\lambda_i = 161$ meV (see main text). ΔE is the driving force, k_{B} is the Boltzmann constant, T is the temperature (= 300 K), $\hbar\omega$ is the high-frequency intramolecular vibrational energy (=165 meV),[6] $S = \lambda_i/\hbar\omega$ is the Huang−Rhys parameter associated to the intramolecular reorganization energy, and l is the number of vibrational quanta included. In the plot of Figure 4 of the main text, we have used the coupling strength of 21 meV and 32 meV for hole transfer and electron transfer, respectively. The energetic parameters (ΔE) we calculated from the EL/sEQE spectra (Table S2).[6, 7, 8]

Supplementary References

1. Causa, M. *et al.* The Fate of Electron–Hole Pairs in Polymer:Fullerene Blends for Organic Photovoltaics. *Nat. Commun.* **7**, 12556 (2016).

2. Fürstenberg, A., Vauthey, E. Ultrafast Excited-State Dynamics of Oxazole Yellow DNA Intercalators. *J. Phys. Chem. B* **111**, 12610-12620 (2007).

3. Vandewal, K., Tvingstedt, K., Gadisa, A., Inganäs, O., Manca, J. V. Relating The Open-Circuit Voltage to Interface Molecular Properties of Donor:Acceptor Bulk Heterojunction Solar Cells. *Phys. Rev. B* **81**, 125204 (2010).

4. Jortner, J. Temperature Dependent Activation Energy for Electron Transfer between Biological Molecules. *J. Chem. Phys.* **64**, 4860-4867 (1976).

5. Marcus, R. A. Relation between charge transfer absorption and fluorescence spectra and the inverted region. *J. Phys. Chem.* **93**, 3078-3086 (1989).

6. Unger, T., Wedler, S., Kahle, F.-J., Scherf, U., Bässler, H., Köhler, A. The Impact of Driving Force and Temperature on the Electron Transfer in Donor–Acceptor Blend Systems. *J. Phys. Chem. C* **121**, 22739-22752 (2017).

7. Liu, X., Li, Y., Ding, K., Forrest, S. Energy Loss in Organic Photovoltaics: Nonfullerene Versus Fullerene Acceptors. *Phys. Rev. Appl.* **11**, 024060 (2019).

8. Qian, D. *et al.* Design Rules for Minimizing Voltage Losses in High-Efficiency Organic Solar Cells. *Nat. Mater.* **17**, 703-709 (2018).
